# Programmed Transformation of Osteogenesis Microenvironment by a Multifunctional Hydrogel to Enhance Repair of Infectious Bone Defects

**DOI:** 10.1002/advs.202409683

**Published:** 2025-01-22

**Authors:** En Xie, Zhangqin Yuan, Qianglong Chen, Jie Hu, Jiaying Li, Kexin Li, Huan Wang, Jinjin Ma, Bin Meng, Ruoxi Zhang, Haijiao Mao, Ting Liang, Lijie Wang, Chaoyong Liu, Bin Li, Fengxuan Han

**Affiliations:** ^1^ Orthopedic Institute Department of Orthopedic Surgery Medical 3D Printing Center The First Affiliated Hospital Changzhou Geriatric hospital MOE Key Laboratory of Geriatric Diseases and Immunology, School of Basic Medical Sciences Suzhou Medical College Soochow University Suzhou Jiangsu 215000 P. R. China; ^2^ Beijing Advanced Innovation Center for Soft Matter Science and Engineering College of Life Science and Technology Beijing University of Chemical Technology Beijing 100029 P. R. China; ^3^ Department of Orthopaedic Surgery The First Affiliated Hospital of Ningbo University Ningbo Zhejiang 315020 P. R. China; ^4^ Sanitation & Environment Technology Institute of Soochow University Ltd. Suzhou Jiangsu 215000 P. R. China

**Keywords:** antimicrobial property, hydrogel, infected bone defects, melatonin carbon dot, microenvironment, osteogenesis

## Abstract

Repair of infectious bone defects remains a serious problem in clinical practice owing to the high risk of infection and excessive reactive oxygen species (ROS) during the early stage, and the residual bacteria and delayed Osseo integrated interface in the later stage, which jointly creates a complex and dynamic microenvironment and leads to bone non‐union. The melatonin carbon dots (MCDs) possess antibacterial and osteogenesis abilities, greatly simplifying the composition of a multifunctional material. Therefore, a multifunctional hydrogel containing MCDs (GH‐MCD) is developed to meet the multi‐stage and complex repair needs of infectious bone injury in this study. The GH‐MCD can intelligently release MCDs responding to the acidic microenvironment to scavenge intracellular ROS and exhibit good antibacterial activity by inducing the production of ROS in bacteria and inhibiting the expression of *secA2*. Moreover, it has high osteogenesis and long‐lasting antimicrobial activity during bone repair. RNA‐seq results reveal that the hydrogels promote the repair of infected bone healing by enhancing cellular resistance to bacteria, balancing osteogenesis and osteoclastogenesis, and regulating the immune microenvironment. In conclusion, the GH‐MCD can promote the repair of infectious bone defects through the programmed transformation of the microenvironment, providing a novel strategy for infectious bone defects.

## Graphical abstract

A multifunctional hydrogel (GH‐MCD) containing MCDs can meet the multi‐stage and complex repair needs of infectious bone injuries by the programmed transformation of the osteogenesis microenvironment. In the early stage, the MCDs kill bacteria by charge effect, inducing bacterial oxidative stress inhibiting bacterial metabolism, and providing a favorable microenvironment for cell growth and sustaining intracellular ROS balance. During the period of bone formation, the MCDs kill intracellular bacteria by enhancing autophagy and regulating the balance of osteogenesis and osteoclastogenesis to promote the repair of infected bone defects.

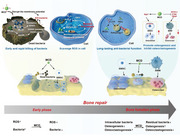

## 1 Introduction

Bone defects caused by various factors are one of the leading causes of morbidity and disability worldwide, with ≈40% of the patients being at risk of relapse and chronic infection.^[^ ^1^, ^2^, ^3^ ^]^ Infection in bone defects remains an intractable complication with a challenging treatment in clinical practice.^[^ ^4^ ^]^ Currently, the clinical treatment of infected bone defects is carried out in two main stages. The first stage involves debridement and the application of antibiotics to control the infection. After controlling the infection, the second stage is to repair the defect with autogenous or allogeneic bone grafts. Although systemic antibiotic therapy effectively relieves the infection, it may produce various serious adverse effects. Autologous or allogeneic bone grafts also face the challenges of donor shortage and immune rejection.^[^ ^5^ ^]^ Although the grafts can accelerate bone repair, the presence of grafts can greatly increase the incidence of bacterial infections.^[^ ^6^ ^]^ Therefore, there is an urgent need for a new material that can promote bone repair and kill bacteria.

The microenvironment of infectious bone defects is complex, which proposes complex and ever‐changing repair requirements for the biomaterials.^[^ ^7^ ^]^ The presence of bacteria is a huge threat to bone repair. After binding and colonizing the bone matrix, bacteria can produce a variety of virulence factors, such as acids and proteases, which could damage the extracellular matrix, and the destruction of the bone extracellular matrix will further facilitate bacterial invasion.^[^ ^8^, ^9^ ^]^ Also, extracellular matrix components interact with osteoblast surface receptors such as integrins, which are important signaling pathways that regulate osteoblast differentiation, maturation, and mineralization. Thus, infection can limit local bone repair capacity.^[^ ^10^ ^]^ The ability of tissue surrounding the implant to defend against bacterial invasion is relatively weak, especially in an unhealthy microenvironment. Notably, infection may arise at any point, even beginning when scaffolds are implanted. However, the initial 4 weeks after implantation are the peak period of infection,^[^ ^11^, ^12^ ^]^ because an osseointegrated interface has not formed yet, and the bactericidal activity of the interface is weak. Even after prolonged antibacterial treatment, residual bacteria may persist within the bone. Moreover, the acidic microenvironment^[^ ^13^ ^]^ at the infected bone defect site is conducive to the release of various virulence factors,^[^ ^12^, ^14^ ^]^ which facilitate bacterial colonization at the implant surface to form biofilms,^[^ ^12^, ^15^ ^]^ eventually resulting in the recurrence of infection. Therefore, it is necessary to develop implant scaffolds with long‐term antibacterial effects to prevent infection until an osseointegrated interface has completely formed. Ideal materials for this type of scaffold should quickly kill bacteria, inhibit biofilm formation in the early stages of infection,^[^ ^16^, ^17^ ^]^ and have sustained antibacterial effects to eliminate residual bacteria along with high osteogenic activity. The specific pathological microenvironment of infectious bone defects is another critical issue. In particular, the microenvironment of infectious bone defects has a low pH (4.5–6.5) and high levels of hydrogen peroxide (H_2_O_2_).^[^ ^16^, ^18^, ^19^, ^20^ ^]^ Reactive oxygen species (ROS) produced by cellular metabolism can act as signaling molecules to regulate many physiological and pathophysiological processes.^[^ ^21^ ^]^ However, excessive production of ROS in the microenvironment of infectious bone injury induces oxidative stress, leading to cellular damage, mitochondrial malfunction, and reduced activity of intracellular antioxidant enzymes.^[^ ^22^ ^]^ Notably, excess ROS poses enormous obstacles to osteogenesis by inhibiting the cell viability of osteoblasts, decreasing alkaline phosphatase (ALP) activity, and decreasing the expression levels of osteogenesis‐related genes.^[^ ^23^ ^]^ In addition, the overproduction of ROS further promotes osteoclastogenesis, stimulates osteoblast activity, and inhibits osteoblast differentiation, which could delay bone repair.^[^ ^24^ ^]^ At the bone/implant interface, the high level of ROS significantly reduces the osteoblastic function of the implant,^[^ ^25^ ^]^ ultimately leading to bone repair failure. Therefore, there is an urgent need to develop a new material that can quickly kill bacteria and improve the pathological microenvironment in the early stages, as well as have long‐lasting antibacterial effects of removing the residual bacteria and high osteogenesis activity in the later bone‐forming phase.

Carbon dots (CDs), a new type of carbon‐based nanomaterial, have attracted extensive attention owing to their small size (<20 nm),^[^ ^26^ ^]^ multiple functional groups (e.g., carboxyl groups, hydroxyl groups, and amines), excellent water solubility, modifiable surface properties, and broad application prospects.^[^ ^26^, ^27^, ^28^, ^29^, ^30^, ^31^ ^]^ CDs have been used in various fields, including cancer treatment,^[^ ^32^, ^33^, ^34^ ^]^ drug delivery,^[^ ^34^ ^]^ bioimaging,^[^ ^35^, ^36^ ^]^ phototherapy, biomedicine, and sensing.^[^ ^28^, ^32^, ^37^ ^]^ In addition, they are emerging materials in the field of antimicrobial treatment.^[^ ^38^ ^]^ At present, very few materials can achieve antibacterial and osteogenesis effects at the same time, and most are combinations of antimicrobial and osteogenic materials, which may increase the complexity of the materials and result in high research and production costs.^[^ ^39^ ^]^ Moreover, many antimicrobial materials are also toxic to the human body. In contrast, CDs have a variety of biological activities and low toxicity, which will inevitably make them shine in infectious bone defect repair. Positively charged CDs can interact with the negatively charged bacterial cell membrane to destroy the integrity of the membrane and cause leakage of intracellular components.^[^ ^40^, ^41^, ^42^, ^43^ ^]^ Owing to their small size, CDs can readily enter bacteria, subsequently interfering with bacterial gene expression and protein synthesis,^[^ ^43^ ^]^ and inhibiting biofilm formation by preventing adhesion and sensing.^[^ ^44^ ^]^ Furthermore, CDs can induce the production of a large amount of ROS in bacteria, leading to oxidative damage. The synergistic effect of multiple antibacterial mechanisms of CDs improves the sterilization efficiency and makes it difficult for bacteria to develop drug resistance. A study showed that CDs extracted from spermidine (CQD_Spds_) had high antimicrobial activity.^[^ ^45^ ^]^ Compared with spermidine, CQD_Spds_ had a smaller size, higher positive charge, and lower minimum inhibitory concentration, killing bacteria more effectively. Li et al. used gentamicin sulfate and diammonium citrate as precursors to synthesize antimicrobial carbon quantum dots (CQDAG) with low drug resistance and anti‐biofilm activity.^[^ ^46^ ^]^ We have previously shown that arginine CDs can distinguish between bacteria and cells. Arginine CDs can kill bacteria by producing excess intracellular ROS and protect cells by increasing the expression of antioxidant proteins.^[^ ^47^ ^]^ In addition to exerting antibacterial effects, CDs have been shown to promote bone regeneration. Ren et al. synthesized novel metformin CDs that promoted osteogenesis in rat bone marrow‐derived mesenchymal stem cells (rBMSCs) under both inflammatory and normal conditions.^[^ ^48^ ^]^ The abovementioned studies suggest that CDs have broad application prospects in treating infected bone defects. In particular, CDs serve as a promising material for modulating the ROS microenvironment, promoting bone formation, and eliminating bacteria, thereby overcoming challenges of addressing complex microenvironments and meeting ever‐changing repair requirements.

As an endogenous hormone, melatonin has strong antioxidant activity,^[^ ^49^ ^]^ and some studies have shown that melatonin can inhibit osteoclast activity and promote bone formation, which makes its application in bone repair quite promising.^[^ ^50^ ^]^ However, the weak antimicrobial effect of melatonin limits its application in infected bone defects.^[^ ^51^ ^]^ New antibacterial activity was demonstrated by preparing carbon dots based on many substances. Therefore, we envisage preparing melatonin as carbon dots to endow melatonin carbon dots (MCDs) with sufficient antimicrobial properties, while still retaining the biological activity of melatonin. In this way, a material with both antibacterial and osteogenic functions was obtained, which improved the microenvironment of bone repair, adapted to the needs of different stages of infectious bone injury repair, and conquered the shortcomings of the current complex treatment methods in the field of infectious bone defects. At the same time, the excellent biocompatibility of carbon dots may also solve the possible toxicity problem of current antimicrobial drugs.

**Scheme 1:**
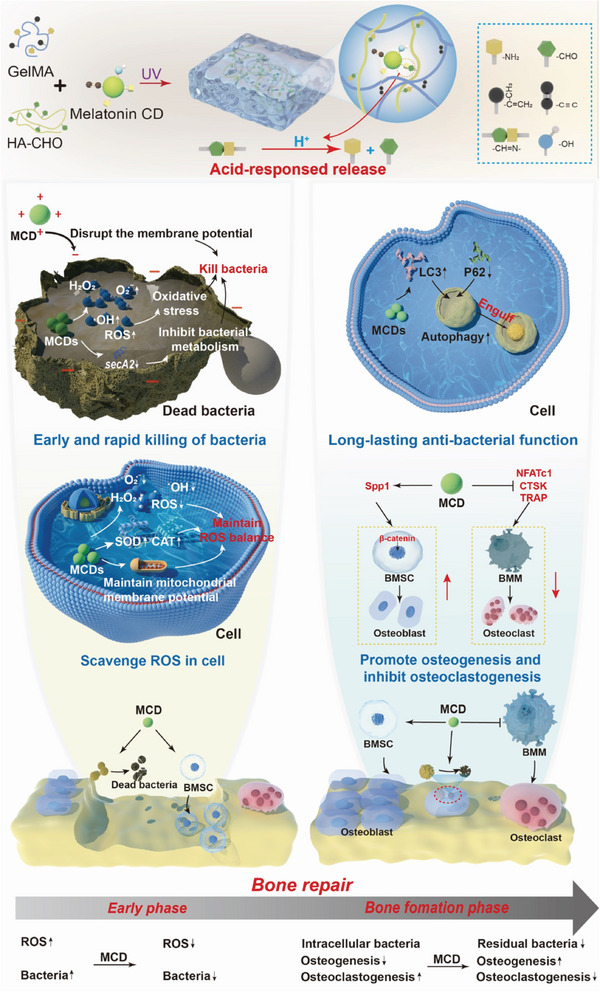
A multifunctional hydrogel (GH‐MCD) containing MCDs can meet the multi‐stage and complex repair needs of infectious bone injuries by the programmed transformation of the osteogenesis microenvironment. In the early stage, the MCDs kill bacteria by charge effect, inducing bacterial oxidative stress and inhibiting bacterial metabolism, and providing a favorable microenvironment for cell growth and sustaining intracellular ROS balance. During the period of bone formation, the MCDs kill intracellular bacteria by enhancing autophagy and regulating the balance of osteogenesis and osteoclast genesis to promote the repair of infected bone defects.

In this study, we propose a novel strategy of programmed transformation of osteogenesis to address the complex microenvironment and meet the multi‐stage and complex repair needs of infectious bone injury (**Scheme** **1**). For this purpose, MCDs were first prepared from melatonin using hydrothermal synthesis, and then incorporated into a GelMA (Gelatin Methacrylate)–oxidized hyaluronic acid hydrogel to form composite hydrogel (GH‐MCD) based on Schiff‐base bond, which can make the MCDs released faster under acidic conditions of infectious bone defects. Both in vitro and in vivo experiments showed that this multifunctional hydrogel GH‐MCD can quickly kill bacteria and inhibit biofilm formation in the early stage, and have long‐lasting antibacterial effects and high osteogenesis activity in the later stage. Moreover, the GH‐MCD could activate β‐catenin/RUNX2 to promote osteogenesis by enhancing the mRNA expression of *spp1*. Our work provides a new strategy for meeting the requirement of the entire critical process of infectious bone defect repair.

## 2 Results

### 2.1 Characterizations of the MCDs

**Figure 1:**
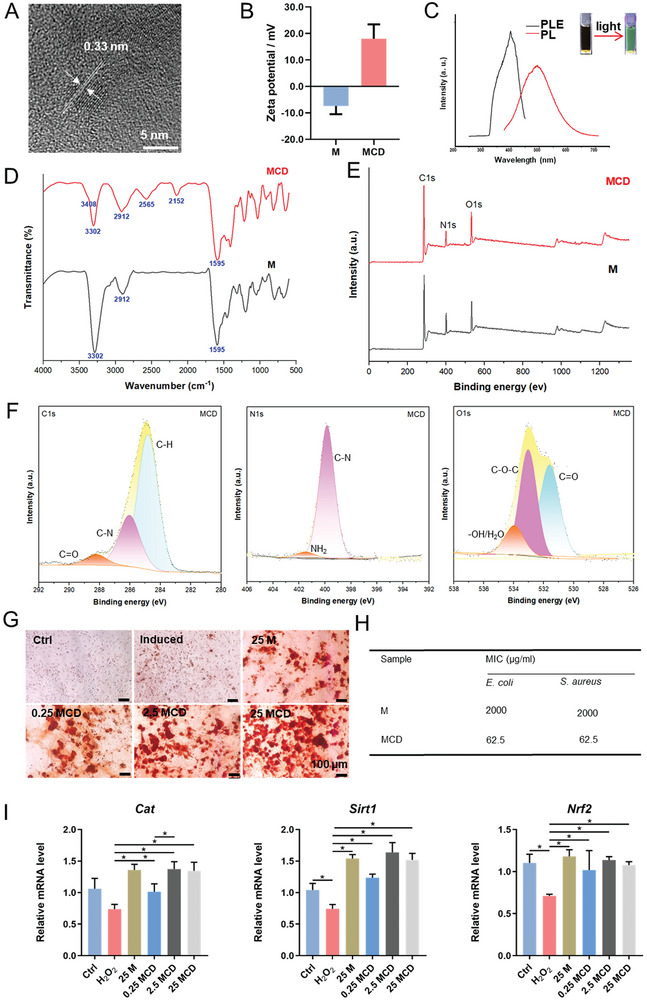
Characterizations of the MCDs. A) Transmission electron microscopy images of the MCDs. B) Zeta potential of the MCDs and melatonin. C) Excitation and emission spectra of the MCDs. D) Fourier transform infrared spectroscopy spectra of the MCDs. E) X‐ray photoelectron spectroscopy results of the MCDs and melatonin. F) C1s, N1s and O1s of the MCDs. G) Alizarin red staining of MCDs and melatonin‐induced BMSCs. H) MIC of the MCDs and melatonin. I) Expression of antioxidant genes in MCDs and melatonin‐treated BMSCs. (^*^ *p* < 0.05).

The MCDs were synthesized by hydrothermal method. As shown in **Figure** **1**A and S1A (Supporting Information), the MCDs were ≈4 nm in diameter, and the lattice distance of MCD was 0.33 nm, corresponding to the (002) plane of graphite carbon. The X‐ray Diffraction (XRD) results showed new peaks ≈2θ = 26°, also corresponding to the 002 plane of graphite carbon (Figure S1B, Supporting Information). The zeta potential results showed that melatonin was negatively charged because it contained amide bonds. Nevertheless, the MCDs were positively charged, which may be attributed to the amide bond hydrolysis at high temperatures to produce an amino group (Figure 1B). The UV absorption spectra showed that the UV absorption peak of melatonin became very broad after being prepared into MCDs (Figure S1C, Supporting Information). The fluorescence spectroscopy showed that the excitation wavelength of the MCDs was 395 nm, and the emission wavelength was 500 nm (Figure 1C). Further, the chemical structure and composition of MCDs were determined using Fourier transform infrared spectroscopy spectra (FTIR) and X‐ray photoelectron spectroscopy (XPS). The MCDs contain many functional groups in melatonin, such as ‐CH_2_ (1485.1 cm^−1^) and ‐N‐C═O (3305.1 cm^−1^). However, the MCDs have some new functional groups, such as ‐OH (2565 cm^−1^), C≡C (2152.3 cm^−1^), and ‐NH_2_ (3408.5 cm^−1^) (Figure 1D). Similar results were also shown in the XPS (Figure 1E). melatonin were shown in XPS, such as C‐H, C‐N, N‐CO, C‐O‐C, etc. (Figure S1D, Supporting Information), and these chemical bonds were preserved in the MCDs. In addition, new amino groups were also generated in the MCDs (Figure 1F).

Next, the biocompatibility of the MCDs was evaluated by cell counting kit‐8 (CCK‐8) assay. The results showed that the MCDs did not produce cytotoxicity until the concentration reached 100 µg mL^−1^, similar to melatonin, proving its excellent biocompatibility (Figure S1E, Supporting Information). The osteogenesis capacity of melatonin and MCDs was then compared. Alizarin red staining showed that MCDs significantly promoted BMSCs calcium deposition compared to melatonin (Figure 1G). ALP staining showed the same results (Figure S2A, Supporting Information). These results were also supported by the expression of genes related to osteogenic differentiation (Figure S2B, Supporting Information). The antimicrobial properties of melatonin and MCDs also differed. The results showed that the MIC of melatonin against *S. aureus* and *E. coli* was 2 mg mL^−1^, while the MIC of MCDs was 62.5 µg mL^−1^ (Figure 1H). Moreover, the mRNA levels of *Cat*, *Sirt1*, and *Nrf2* in MCDs‐treated BMSCs were similar to those in the melatonin‐treated groups, suggesting that the MCDs retained the antioxidant properties of melatonin (Figure 1I). According to intracellular ROS clearance efficiency results, the MCDs have a stronger antioxidant function than melatonin (Figure S2C, Supporting Information). At a concentration of 2.5 µg mL^−1^, MCDs achieved the same effect as melatonin at 25 µg mL^−1^. The above results showed that the biological function of melatonin was preserved or even greatly enhanced after being prepared into the MCDs.

### 2.2 Characterizations of the GH‐MCD Hydrogels

**Figure 2:**
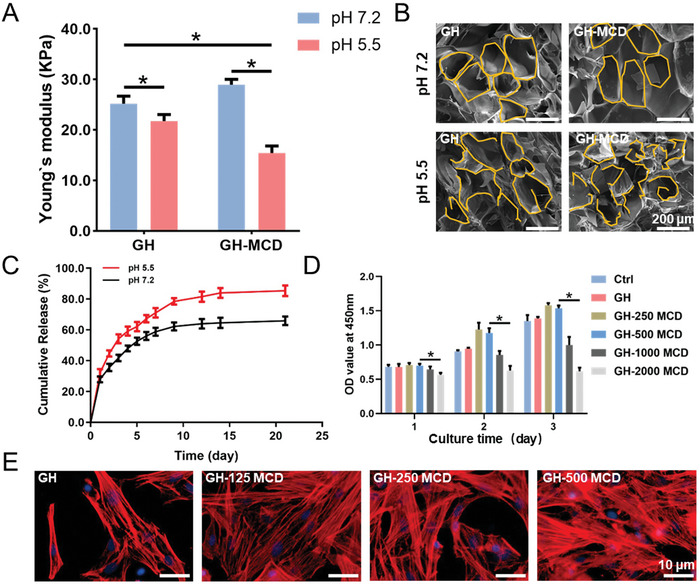
Characterizations of the GH‐MCD hydrogels. A) Compressive modulus. B) SEM images of GH‐MCD hydrogels in neutral and acidic solutions. C) Release curve of MCDs from GH‐MCD hydrogels in neutral and acidic solution, respectively. D) CCK‐8 assay of the BMSCs treated with the hydrogels. E) Cytoskeleton staining of cells cultured on the GH‐MCD hydrogels containing different amounts of MCDs. (^*^ *p* < 0.05).

The mechanical strength of the composite hydrogel was first tested. The results showed that the compressive modulus of GH hydrogel increased from 25.2 ± 1.5 to 29.0 ± 1.1 kPa after being mixed with MCD. The compressive modulus of GH‐MCD hydrogels was lower than that of GH hydrogels after overnight immersion in acidic PBS (pH 5.5). This was attributed to the break of Schiff base bonds being under acidic conditions. (**Figure** **2**A). The images of scanning electron microscopy (SEM) showed that both hydrogels formed a loose and porous structure under neutral conditions. In contrast, the GH‐MCD composite hydrogel showed a more disrupted pore structure in an acidic medium (Figure 2B). It was found that the GH‐MCD hydrogels could release MCDs faster under acidic conditions than neutral conditions, reaching ≈80% at 21 days (Figure 2C).

Next, we conducted a CCK‐8 assay to explore the biocompatibility of the GH‐MCD hydrogels. As shown in Figure 2D, GH‐250 MCD and GH‐500 MCD hydrogels don't show cytotoxicity compared to the Ctrl group. Cell proliferation was not affected until the concentration of the MCDs was increased to 1 mg mL^−1^ (Figure 2D). Rat BMSCs were seeded on the surface of different hydrogels to observe the adhesion and spreading of cells. The results of skeleton staining showed that the GH hydrogel was conducive to cell adhesion and spreading. BMSCs could grow on the surface of GH‐MCD hydrogels, and the number of filamentous pseudopods of BMSCs was significantly higher than those on the GH hydrogels (Figure 2E). These results indicated that MCDs released from the GH‐MCD hydrogels still maintain good biological activity and could be used as a suitable bone repair material.

### 2.3 Antioxidant Properties of the GH‐MCD Hydrogels

**Figure 3:**
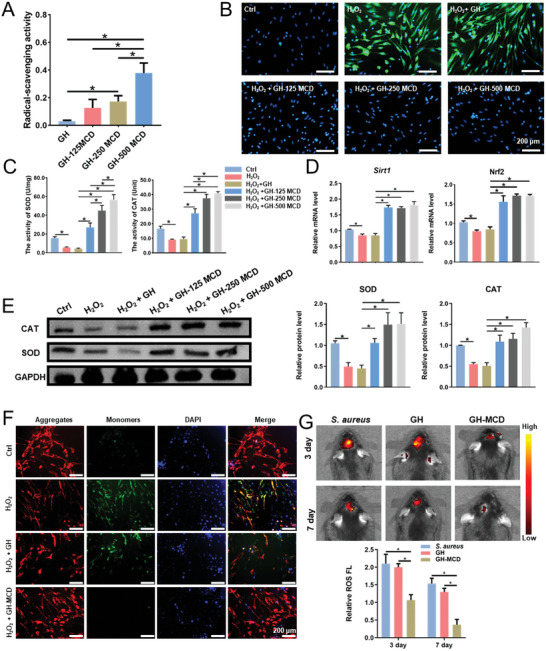
Antioxidant properties of the GH‐MCD hydrogels. A) DPPH radical scavenging rate of the hydrogels. B) Fluorescence images of ROS‐positive BMSCs treated with H_2_O_2_. C) SOD and CAT activity of BMSCs cultured on the hydrogels. D) Expression of antioxidant genes of BMSCs cultured on the hydrogels. E) Expression of antioxidant protein of BMSCs cultured on the hydrogels and their quantitative analysis. F) The mitochondrial membrane potential of BMSCs cultured on the hydrogels. G) In vivo removal of ROS. (^*^ *p* < 0.05).

Removing excess ROS from the site of bone damage may accelerate bone healing. As shown in **Figure** **3**A, the free radical scavenging rate of GH‐MCD hydrogels gradually increased with the concentration of MCDs. The exogenous free radical scavenging rate in the GH‐500 MCD group was ≈35%. To examine the effects of the GH‐MCD hydrogels on intracellular ROS, we added H_2_O_2_ and GH‐MCD to the cell culture medium simultaneously and performed DCFH‐DA staining after 1 day. The results showed that the GH‐MCD hydrogels significantly scavenged intracellular ROS, and protected cells against oxidative damage (Figure 3B), and increased the production of SOD and CAT in cells (Figure 3C). Furthermore, the effects of the GH‐MCD hydrogels on intracellular antioxidant genes were evaluated. The results of qPCR showed that after 1 day of treatment with H_2_O_2_ and hydrogels, the GH‐MCD hydrogels promoted the expression of *Sirt1* and *Nrf2* compared with GH hydrogels (Figure 3D). The results of Western blotting were consistent with those of qPCR. In particular, GH‐MCD hydrogels significantly increased the protein expression of SOD and CAT (Figure 3E). Subsequently, the mitochondrial membrane potential, which is a major factor driving ROS production, was detected using the JC‐1 probe. The GH‐500 MCD group showed the most significant effects and was selected as the experimental group (GH‐MCD) for subsequent experiments. The GH‐MCD hydrogels maintained the equilibrium of mitochondrial membrane potential, indicating that they may regulate ROS by modulating mitochondrial homeostasis (Figure 3F).

The removal of ROS by the GH‐MCD hydrogels was validated in mouse models of skull defects. On day 3, the fluorescence intensity of the *S. aureus* and GH hydrogel groups was extremely strong, indicating that a large amount of ROS was generated, whereas that of the GH‐MCD group was substantially weak. After 7 days, the *S. aureus* and GH hydrogel groups retained the strong fluorescence intensity. In contrast, no distinguishable fluorescence intensity was observed in the GH‐MCD group, indicating that most ROS were removed from the defects (Figure 3G).

### 2.4 Antibacterial Effects of the GH‐MCD Hydrogels

**Figure 4:**
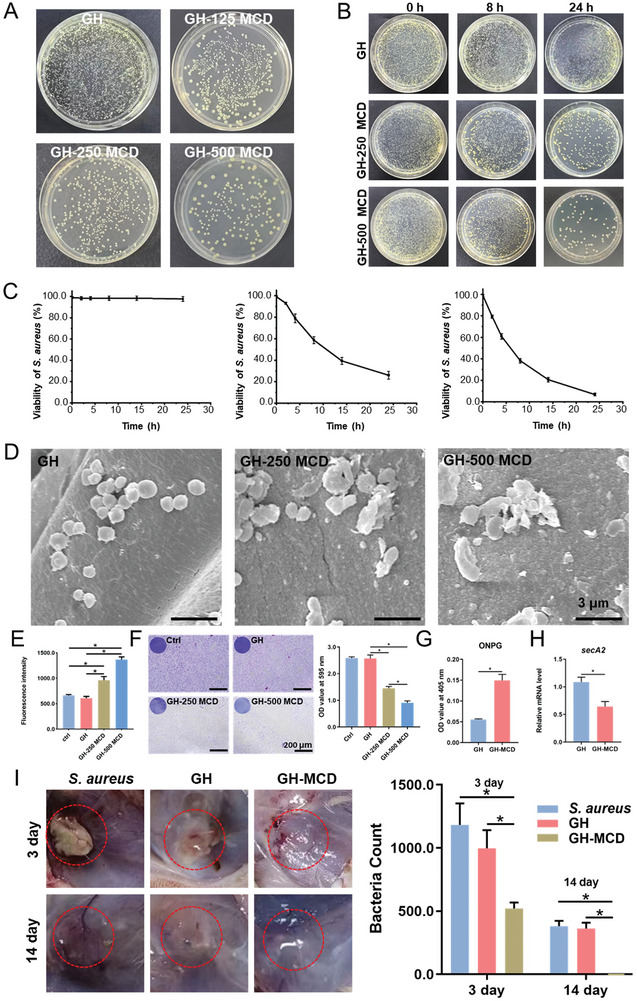
Antibacterial properties of the GH‐MCD hydrogels. A) Colonies of *S. aureus* cultured on the hydrogels. B) Colonies of *S. aureus* cultured on different hydrogels at different time points and C) Quantification of viability of *S. aureus*. D) Morphology of *S. aureus* cultured on the surface of the hydrogels. E) Quantity of ROS generated in bacteria cultured on the hydrogels. F) Microscopic observation of biofilms by crystal violet staining and quantitative analysis of crystal violet staining. G) ONPG assay. H) Gene expression related to energy metabolism in bacteria. I) Colonies of *S. aureus* counted on the hydrogels taken out from femoral critical‐sized defects in rats at 3 and 7 days after implantation. (^*^ *p* < 0.05).

The *S. aureus* was co‐cultured with hydrogels for 24 h. The standard plate counting assay showed that after 24 h, the number of bacteria surviving on the GH hydrogels did not decrease. However, the bacterial survival rates in the GH‐125 MCD and GH‐250 MCD groups were ≈60% and 30%, respectively. Notably, the GH‐500 MCD group had the lowest bacterial survival rate, ≈10% (**Figure** **4**A; Figure S3A, Supporting Information). The GH‐125 MCD hydrogel had insufficient antibacterial effects and was excluded from subsequent experiments. AO/EB staining showed that the number of dead bacteria on the GH‐250 MCD and GH‐500 MCD hydrogels was significantly increased (Figure S3B, Supporting Information). The results of antimicrobial kinetics were consistent with those tested by the standard plate counting assay. In particular, the GH‐500 MCD hydrogels killed ≈90% of the bacteria within 24 h (Figures 4B,C). SEM images showed that in the absence of MCDs, *S. aureus* exhibited a spherical and smooth structure. However, in the presence of MCDs, the bacterial structure was disrupted, with the extent of shrinkage and rupture depending on the concentration of MCDs (Figure 4D). Next, the bactericidal effect of the GH‐MCD hydrogel on *E. coli* was also explored. The results of the standard plate counting assay showed that the number of *E. coli* grown on the surface of the GH‐MCD hydrogel was significantly reduced after one day (Figure S3C, Supporting Information). The SEM results showed that the *E. coli* grown on the surface of the hydrogel up to the degree of fragmentation was more obvious with the increase in the concentration of the MCDs (Figure S3D, Supporting Information). These results indicate that the GH‐MCD hydrogel has a certain antibacterial effect on Gram‐negative bacteria. Furthermore, ROS activity was detected to obtain detailed mechanistic insights into the antibacterial activity of the GH‐MCD hydrogels. The results showed that bacterial ROS accumulation was lower in the GH group than in the GH‐MCD group. In GH‐MCD hydrogels, ROS accumulation increased with an increase in the concentration of MCDs, eventually resulting in bacteria death (Figure 4E). Bacterial resistance to antibiotics and host immune responses increases after the formation of biofilms. To examine the effects of the GH‐MCD hydrogels on biofilm formation, bacteria were cultured on the GH‐MCD hydrogels for 2 days and crystal violet staining was subsequently performed. The results showed almost no damage to biofilms in the GH group; however, the damaging effects of the GH‐MCD hydrogels on biofilms gradually increased with an increase in the concentration of MCDs. In particular, the biofilm formation rate in the GH‐500 MCD group was less than half of that in the control group (Figure 4F). An ONPG assay was performed to investigate the extent of damage to the bacterial membrane after hydrogel treatment. The results showed that the permeability of the bacterial membrane was significantly increased after treatment with the GH‐MCD hydrogel, indicating that the bacterial membrane was seriously damaged (Figure 4G). In addition, we also examined the expression of genes related to bacterial metabolism. We found that key genes for bacterial energy metabolism, such as *secA2*, were significantly downregulated in the GH‐MCD group (Figure 4H). These results indicate that the GH‐MCD hydrogels play a bactericidal role by destroying the integrity of bacterial membranes and regulating bacterial metabolism. We also found that the GH‐MCD hydrogels induce the M1 polarization of macrophages (Figure S3F, Supporting Information), which means that in the early stage of implantation, the GH‐MCD‐induced M1‐polarized macrophages can exert a powerful phagocytic effect to kill bacteria and effectively control infection.

The antibacterial effects of the GH‐MCD hydrogels were evaluated in rats with infectious femoral condylar defects. After 3 days, abscess cavity formation was observed at the defect site in the control and GH hydrogel groups but not in the GH‐MCD group. Rat femur samples were rinsed with PBS, and a certain amount of PBS was absorbed to conduct the standard plate counting assay. The results showed that the number of bacteria was significantly reduced in the GH‐MCD group. After 2 weeks, all bacteria were eliminated, indicating that the GH‐MCD hydrogels had excellent in vivo bactericidal effects (Figure 4I; Figure S3E, Supporting Information).

### 2.5 In Vitro Osteogenic Activity of GH‐MCD Hydrogels

**Figure 5:**
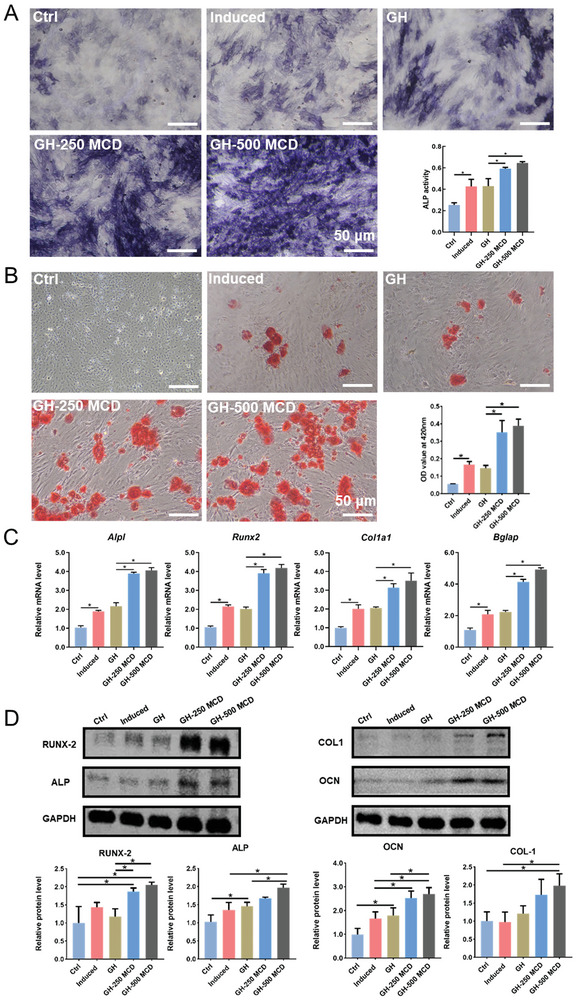
In vitro osteogenesis of BMSCs cultured on the GH‐MCD hydrogels. A) ALP staining and its quantification. B) Alizarin red staining and its quantification. Expression of osteogenesis‐related (C) genes and (D) proteins of the BMSCs cultured on different hydrogels and quantitative analysis. (^*^ *p* < 0.05).

Concerning the effects of CDs on bone metabolism, animal and human studies have shown that melatonin‐modified biomaterials can promote fracture repair.^[^ ^52^, ^53^, ^54^ ^]^ Therefore, we evaluated the osteogenic activity of GH‐MCD hydrogels in vitro. As shown in **Figure** **5**A, GH‐250 MCD and GH‐500 MCD hydrogels significantly promoted the expression of ALP in BMSCs, with GH‐500 MCD hydrogels having the strongest effect. These results indicate that MCDs can promote the osteogenic differentiation of BMSCs in the early stage. Alizarin red staining showed similar results. BMSCs in the osteogenic induction and GH groups had fewer and smaller calcium nodules, whereas those treated with GH‐250 MCD or GH‐500 MCD hydrogels had more calcium deposition. These results indicate that GH‐MCD hydrogels have long‐term beneficial effects on the osteogenic differentiation of BMSCs (Figure 5B).

Furthermore, the expression of osteogenesis‐related genes and proteins was evaluated in BMSCs treated with composite hydrogels. Compared with GH hydrogels, GH‐MCD hydrogels significantly increased the expression of intracellular osteogenesis‐related genes, such as *Alpl*, *Runx2*, *Col1a1*, and *Bglap* (Figure 5C), which is consistent with the results of ALP and alizarin red staining. In addition, GH‐MCD hydrogels significantly promoted the expression of intracellular osteogenesis‐related proteins including RUNX2, OPN, COL1, and OCN (Figure 5D). Altogether, these results suggested that GH‐MCD hydrogels effectively promoted the osteogenic differentiation of BMSCs. Due to its potent osteogenic activity, the GH‐500 MCD hydrogel was selected for subsequent animal experiments.

### 2.6 Bone Regeneration Induced by the GH‐MCD Hydrogels in Infectious Bone Defects

**Figure 6:**
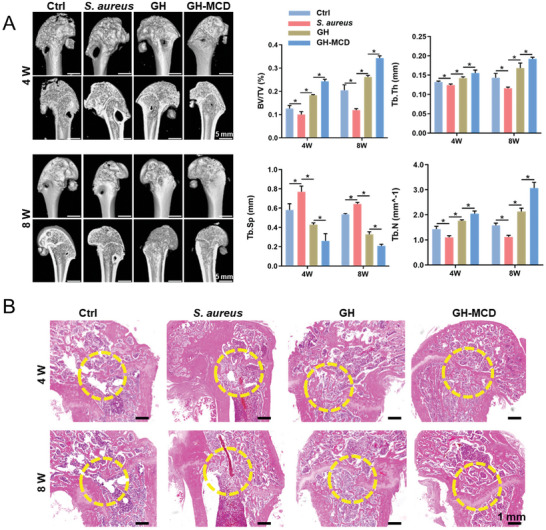
Repair of rat infectious bone defects by the GH‐MCD hydrogels. A) Micro‐CT images of harvested femurs and quantitative analysis. B) H&E staining images. (^*^ *p* < 0.05).

To evaluate the bone repair ability of the GH and GH‐MCD hydrogels, the hydrogels were implanted into infected bone defects of SD rats. After 4 weeks of surgery, femur samples were collected and examined via micro‐computed tomography (micro‐CT). The defects in the bacterial group (*S. aureus*) were more pronounced than those in the simple bone defect group (Ctrl). Some new bone tissues were observed in the GH hydrogel group, indicating that GH hydrogels had a weak ability to repair infectious bone defects. GH provides a means of signal communication and nutrient exchange between cells, thus promoting bone repair.^[^ ^55^ ^]^ In the GH‐MCD group, new bone tissues were completely covering the defect site and integrated with surrounding bone tissues. After 4 weeks, the bone volume fraction (BV/TV) in the GH‐MCD group reached ≈25%. In addition, the trabecular bone thickness (Tb. Th) and number (Tb. N) were highest, and trabecular separation (Tb. Sp) was lowest in the GH‐MCD group. After 8 weeks, the bone surface was almost completely repaired in the GH‐MCD group (**Figure** **6**A). Consistently, H&E staining showed that the GH‐MCD hydrogels promoted bone formation (Figure 6B).

### 2.7 In Vivo Biosafety and Stability of the GH‐MCD Hydrogels

The in vivo biosafety and stability of the GH‐MCD hydrogels were assessed by the mouse subcutaneous model. As shown in the H&E staining images, the visceral sections of mice with subcutaneously embedded hydrogel did not show obvious pathological changes or histomorphological abnormalities compared to normal mice in the Ctrl group, and no side effects or acute pathological systemic toxicity were observed (Figure S4A, Supporting Information). In addition, the GH‐MCD hydrogels demonstrated in vivo degradation (Figure S4B, Supporting Information).

### 2.8 Osteogenesis Mechanism of the GH‐MCD Hydrogels

**Figure 7:**
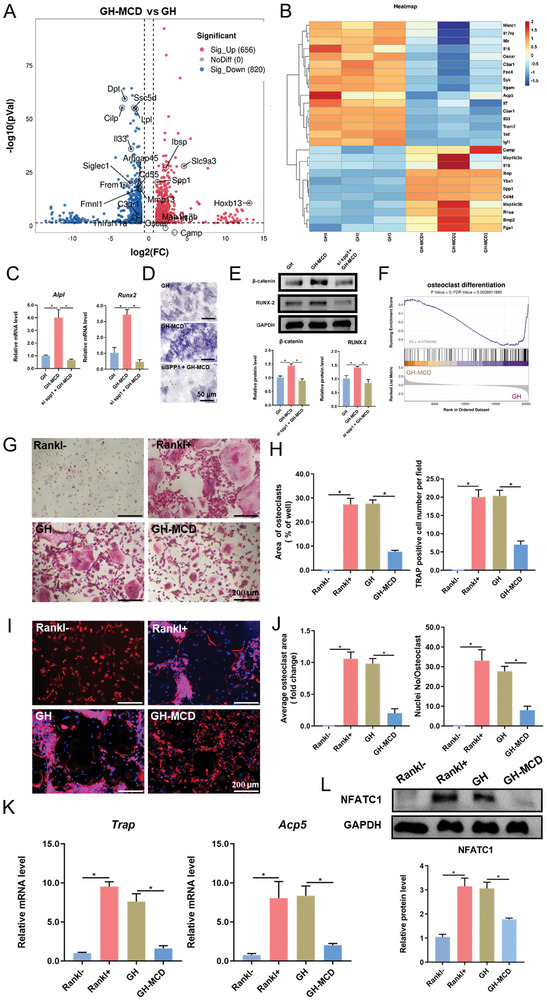
Exploration of the osteogenesis mechanism of the GH‐MCD hydrogels. A) Volcano plot showing differentially expressed genes between GH‐MCD and GH groups. B) Heat map of some genes. C) Gene expression of *Alpl* and *Runx2* of the BMSCs at 5 days. D) ALP staining of BMSCs at 5 days. E) Protein expression of RUNX‐2 and β‐catenin at 5 days before and after Spp1 being knocked down and quantitative analysis. F) Gene set enrichment analysis. G) TRAP staining. H) Quantitative analysis of TRAP staining. I) Podosome belt formation assay. J) Quantitative analysis of Podosome belt formation assay. K) Genes and L) protein expression during osteoclastogenesis of the BMMs at 7 days. (^*^ *p* < 0.05).

To determine the mechanism through which GH‐MCD hydrogels induced osteogenic differentiation of BMSCs, RNA‐seq was performed on day 5 of culture. A heatmap and volcano plot were generated to visualize differentially expressed genes (DEGs) between the GH‐MCD and GH groups (**Figures** **7**A,B; Figure S5A, Supporting Information). Most DEGs were associated with inflammatory responses, osteoclast differentiation, osteoblast differentiation, and complement and coagulation cascades. KEGG and GO enrichment analyses revealed consistent results. Compared with the GH hydrogels, the GH‐MCD hydrogels had significantly lower immunogenicity, as they downregulated immune‐related pathways, including hematopoietic cell lineage‐related pathways, complement and coagulation cascades, platelet activation, natural killer cell‐mediated cytotoxicity and B‐cell receptor signaling, which provided a relatively stable microenvironment for bone regeneration. Furthermore, the GH‐MCD hydrogels increased the activity of genetic information processing‐related pathways, such as RNA polymerase‐related pathways, aminoacyl tRNA biosynthesis, base excision repair, homologous recombination, ribosome biogenesis in eukaryotes, RNA transport, and mismatch repair, which play a crucial role in cell proliferation and differentiation (Figures S5B,C, Supporting Information). We also compared GH‐MCD with GH‐M and found that GH‐MCD was superior to GH‐M in the abovementioned aspects (Figures S5D,E, Supporting Information).

Osteogenic differentiation and bone formation are complex processes involving multiple signaling pathways. Although GH‐MCD hydrogels did not upregulate genes involved in classical pathways such as Wnt, Notch, and MAPK, they upregulated the *Spp1* mRNA expression greatly, as well as *Pgs1*, *RhoA*, *Bmp2*, *Cd44* linking to BMSC osteogenic differentiation, and significantly downregulated the osteoclast differentiation‐related genes, such as *Trem2*, *Itgam*, *Syk*, *Sirpα* and *Tyrobp* (Figures 7A,B).

Considering that GH‐MCD hydrogels significantly increased *Spp1* and *Spp1* played a key role in osteogenic differentiation,^[^ ^31^ ^]^ we guessed that GH‐MCD hydrogels were likely to promote osteogenic differentiation by promoting the expression of *Spp1*. To investigate whether osteogenic SPP1 drives BMSCs osteogenic differentiation by GH‐MCD, we employed an RNAi approach and selected siSPP1(751) as the most effective siRNA (Figure S5F, Supporting Information). It was shown that BMSCs treated with GH‐MCD hydrogels produced more SPP1 and exhibited enhanced osteogenic differentiation. After treatment with siSPP1, the osteogenic differentiation reduced sharply with much lower osteogenic‐related gene *Alpl* and *Runx2* expression, and ALP staining showed similar results (Figures 7C,D). Next, we verified whether wnt/β‐catenin, a classical pathway that promotes bone differentiation, is also regulated by *Spp1*. The results showed that in the presence of GH‐MCD hydrogel, β‐catenin activation and the expression of RUNX‐2 were increased. However, after *Spp1* was knocked down, β‐catenin activation and RUNX‐2 expression were significantly reduced, suggesting that GH‐MCD hydrogel can regulate the RUNX‐2 and wnt/β‐catenin pathway through the positive feedback of *Spp1* and thus regulate osteogenesis (Figure 7E).

GSEA showed that GH‐MCD hydrogels downregulated the osteoclast differentiation pathway compared to GH and GH‐M groups (Figure 7F; Figure S5G, Supporting Information). TRAP staining and podosome belt formation assay were performed to examine the effects of GH‐MCD hydrogels on osteoclast formation. Compared with the GH‐MCD group, the induced group (RANKL+) had a significantly larger area of osteoclasts and a higher number of nuclei (Figures 7G,H). The podosome belt formation assay results were consistent with TRAP staining results (Figures 7I,J). Furthermore, the expression of osteoclast differentiation‐related genes (*Trap* and *Acp5*) and proteins (NFATC1) was examined to verify the inhibitory effects of GH‐MCD hydrogels on osteoclast differentiation. The results showed that the expression of *Trap*, *Acp5*, and NFATC1, was significantly higher in the induced (RANKL+) and GH groups than that in the control group (RANKL‐), whereas that in the GH‐MCD group was significantly decreased and similar to the control group (Figures 7K,L). These results indicate that GH‐MCD hydrogels effectively inhibit osteoclast differentiation.

Altogether, GH‐MCD hydrogels promoted osteogenesis by upregulating *Spp1* and suppressed osteoclast differentiation by downregulating *Trap*, *Acp5*, and NFATC1, eventually promoting bone repair.

### 2.9 Antimicrobial Mechanism of the GH‐MCD Hydrogels

**Figure 8:**
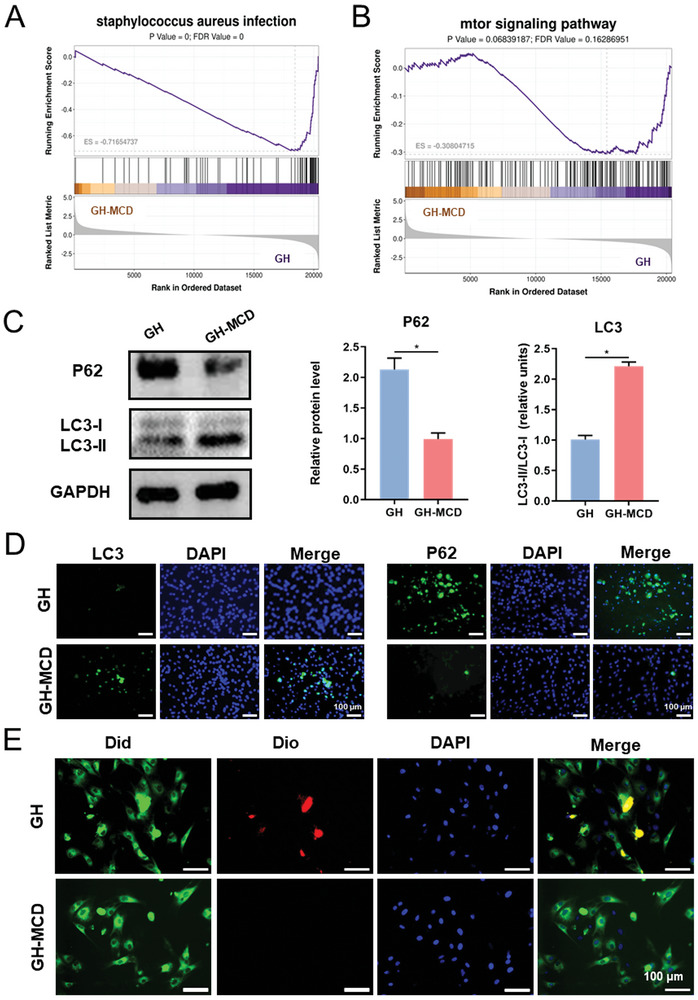
Exploration of the antimicrobial mechanism of the GH‐MCD hydrogels. A, B) Gene set enrichment analysis. C) Protein expression of BMSCs related to autophagy. D) Immunofluorescence staining of autophagy‐related proteins. E) Fluorescence pictures of co‐cultured cells and bacteria. (^*^ *p* < 0.05).

RNA‐seq (**Figure** **8**A) showed that the expression of bacterial receptors and major complement genes was significantly reduced on BMSCs treated with the GH‐MCD hydrogels, which alleviated infection, attenuated inflammatory responses, and enhanced cellular defense against bacteria. In addition, the GH‐MCD hydrogels downregulated the mTOR signaling pathway (Figure 8B), promoting autophagy.^[^ ^56^ ^]^ Even when compared to the GH‐M, the GH‐MCD was superior to GH‐M in inhibiting S. aureus infection and the mTOR pathway (Figure S5H, Supporting Information). Autophagy plays an important role in the clearance of intracellular bacteria,^[^ ^57^ ^]^ decreasing the susceptibility of cells to repeated bacterial infections. As shown in Figure 7B, the GH‐MCD hydrogels upregulated the ratio of protein LC3‐II to LC3‐I, positively correlated with autophagy. They downregulated the P62, negatively correlated with autophagy (Figure 8C). Immunofluorescence staining revealed consistent results (Figure 8D), validating that the GH‐MCD hydrogels promoted autophagy. Subsequently, BMSCs and bacteria were co‐cultured to examine the ability of the GH‐MCD hydrogels to remove intracellular bacteria. A large number of live bacteria were observed in GH hydrogel‐treated BMSCs. In contrast, no live bacteria were observed in the GH‐MCD hydrogel‐treated BMSCs, indicating that the MCDs either exerted strong antibacterial effects or helped the cells to eliminate intracellular bacteria (Figure 8E).

## 3 Discussion

Infected bone defect is a therapeutic challenge in clinical practice owing to its complex microenvironment. After the initiation of bone repair is triggered by an inflammatory response, a controlled and appropriate level of inflammation is essential for subsequent bone reconstruction. However, infection is a persistent stimulus that aggravates inflammation at the site of bone injury, leading to complications such as fibrosis and impaired bone repair.^[^ ^58^, ^59^, ^60^ ^]^ Notably, the inflammatory response is strongly associated with the overproduction of

ROS.^[^ ^61^, ^62^ ^]^ ROS can not only induce oxidative damage, apoptosis, and necrosis^[^ ^63^ ^]^ but also exacerbate inflammation.^[^ ^64^, ^65^, ^66^ ^]^ In addition, excess ROS poses a significant barrier to osteogenesis by inhibiting the viability of osteoblasts, decreasing the activity of ALP, and downregulating osteogenesis‐related genes.^[^ ^23^ ^]^ Therefore, regulating ROS homeostasis is critical to preventing excessive inflammation and promoting bone repair. The early stage of infectious bone injury, when an osseointegrated interface has not yet been formed and resistance to infection is very weak, is considered the peak period of infection.^[^ ^11^, ^67^ ^]^ In the microenvironment of infectious bone defects, a large amount of carbon dioxide is exuded owing to dead or dying cells. In addition, the lack of blood supply to defective segments decreases oxygen levels, leading to the accumulation of lactic acid,^[^ ^68^ ^]^ and excess ROS can activate osteoclasts to secrete a large amount of hydrogen ions (H^+^).^[^ ^68^, ^69^ ^]^ The complex interplay of these factors leads to the formation of a local acidic extracellular microenvironment at the defect site. Under acidic conditions, osteoblast activity is decreased, whereas osteoclast activity is increased,^[^ ^70^ ^]^ leading to enhanced bone resorption, further delaying bone repair. Moreover, the acidic microenvironment of infected bone defects promotes the release of various bacterial virulence factors,^[^ ^12^, ^14^ ^]^ which facilitate colonization and biofilm formation on the implant surface.^[^ ^12^, ^15^ ^]^ These bacteria may remain inside the bone even after long‐term antibiotic treatment, eventually triggering the recurrence of infection.

Given the complex microenvironment of infectious bone defect, it is necessary to fully transform the microenvironment of infectious bone injury, rather than just focusing on antibacterial or bone‐promoting effects. In clinical practice, debridement and the application of antibiotics to control the infection is a conventional method.^[^ ^5^ ^]^ Besides the side effects of antibiotics, this approach does not directly promote bone repair. After controlling the infection, the second stage is to repair the defect with autogenous or allogeneic bone grafts. However, the use of grafts can greatly increase the incidence of bacterial infections.^[^ ^6^ ^]^ Therefore, there is an urgent need for a material with simple composition but powerful functionality. Compared with composite materials that integrate different chemical compositions, the CDs with simple composition, multiple bioactivities, and low toxicity, show great potential in infectious bone repair.^[^ ^26^ ^]^ the CDs can retain and enhance the properties of their precursors and may possess intrinsic properties. Our previous study synthesized a material with excellent antibacterial and osteogenic properties by converting arginine into arginine CDs.^[^ ^47^ ^]^ Melatonin has antioxidant function and has been reported to enhance the osteogenic differentiation of stem cells.^[^ ^71^ ^]^ In addition, it can protect human mesenchymal stem cells by inducing interleukin‐1β‐induced inflammatory responses^[^ ^72^ ^]^ and exert potent antibacterial effects.^[^ ^73^ ^]^ However, the direct antibacterial effects of melatonin may occur only at very high concentrations, which hinders its practical application. Given the properties of the CDs, melatonin's antibacterial and osteogenesis properties may be significantly enhanced by integrating it with the CDs. Therefore, the MCDs may improve the pathological microenvironment, exert antibacterial effects, and promote bone regeneration to effectively treat infectious bone defects.

In this study, the MCDs have good antibacterial, antioxidant, and osteogenesis abilities, greatly simplifying the composition of a multifunctional material. Further, a multifunctional hydrogel (GH‐MCD) containing the MCDs was developed to meet the multi‐stage and complex repair needs of infectious bone injury. For this purpose, melatonin was used to synthesize the MCDs, which were expected to exhibit efficient antioxidant, antibacterial, and osteogenesis properties. The size of CDs is generally in the range of a few nanometers.^[^ ^26^ ^]^ In this study, our MCDs are ≈4 nm in diameter. It has been reported that the antibacterial activity of CDs would increase with their sizes decrease, because they are more likely to enter bacteria to play a role, such as destroying the DNA structure of bacteria.^[^ ^74^ ^]^ At this size, we found that different concentrations also significantly impact its bioactivity. Hence, we have selected the optimal conditions under these conditions. In the future, we will further investigate the impact of different sizes on their bioactivity. The disruption of the bacterial membrane structure is an important mechanism for antimicrobial drugs. The cell membrane inside the cell wall is an important barrier to protect the internal environment of bacteria. Melatonin is negatively charged, whereas MCDs are positively charged due to ‐NH_2_ functional groups. The positively charged MCDs can bind to the negatively charged bacterial cell membrane, disrupting the balance of bacterial membrane potential, disrupting the bacterial membrane, and leading to intracellular leakage. The ONPG assay also supports this result. When the MCDs were added to hydrogels prepared using GelMA and HA‐CHO, the ─NH_2_ groups of the MCDs formed a Schiff base bond with the ─CHO groups of the hydrogels.^[^ ^75^ ^]^ This interaction enabled the GH‐MCD hydrogels to respond to the acidic microenvironment of infectious bone defects and release MCDs early and rapidly to exert bactericidal effects. In vivo experiments showed that the bacterial survival rate was substantially decreased in the GH‐MCD group after 3 days of implantation. No live bacteria were observed after 14 days.

Some antimicrobial materials have also been applied in repairing infected bone defects, such as combining metal nanoparticles or cationic antimicrobial materials with calcium phosphate materials, which have also achieved good performances.^[^ ^39^ ^]^ However, these antimicrobial agents are poorly biocompatible and must be combined with materials for osteogenic activity, which also causes trouble in material preparation. The CDs, however, have excellent biocompatibility and a simple preparation method, as the MCDs can be synthesized by the simple hydrothermal method. The in vivo biocompatibility of carbon dots has been extensively validated. Han et al. used their NIR‐CPDs for in vivo applications. The NIR‐CPDs were all injected intravenously into mice and their metabolic behavior was observed. All carbon dots were excreted from the mice via feces, indicating a typical hepatobiliary clearance pathway.^[^ ^76^ ^]^ Some studies have also shown that small‐sized carbon dots can be cleared via the kidneys. For example, Gao et al. prepared NIR‐CDs for multimodal imaging‐guided therapy.^[^ ^77^ ^]^ Compared with composite materials combining different components, the CDs with simple composition but multiple bioactivities, show great potential in infectious bone repair. Moreover, the easy surface modification of CDs also has a great potential for obtaining new biological functions.^[^ ^78^, ^79^ ^]^ These results highlight that the GH‐MCD hydrogels hold great promise in antibacterial therapy.

We have previously demonstrated that regulating ROS levels in the microenvironment of bone defects and increasing the expression of antioxidant proteins can effectively promote bone repair.^[^ ^23^ ^]^ In this study, the GH‐MCD hydrogels were found to retain the strong antioxidant effects of melatonin,^[^ ^55^ ^]^ which enabled them to scavenge exogenous free radicals, upregulate intracellular antioxidant enzymes to scavenge excessive intracellular ROS, and restore mitochondrial membrane potential in BMSCs. In addition, the GH‐MCD hydrogels decreased the levels of antioxidant enzymes and promoted the production of large amounts of ROS in bacteria, eventually leading to their death. These findings suggest that the MCDs can distinguish between bacterial and mammalian cells and play protective roles accordingly. In addition, we also found that the MCDs affect bacterial growth and metabolism. SecA2 is an ATPase of the classical bacterial Sec secretion system essential for bacterial growth.^[^ ^80^, ^81^ ^]^ In our study, the MCDs can significantly inhibit the mRNA expression of *SacA2*, leading to bacterial metabolic imbalance and death.

RNA‐seq was performed to investigate how the GH‐MCD hydrogels promoted the osteogenic differentiation of BMSCs in vitro. The major DEGs in the GH‐MCD hydrogel‐treated BMSCs were associated with the inflammatory response, osteoclast differentiation, osteoblast differentiation, autophagy, and immune regulation. The balance between bone formation and resorption is essential for maintaining local bone mass and reconstructing normal bone tissue during bone repair. In infectious bone defects, the pathogenic bacteria directly promote the secretion of RANKL from osteoblasts and decrease the expression of OPG, which in turn promotes osteoclast differentiation.^[^ ^82^ ^]^ Moreover, excessive accumulation of pro‐inflammatory factors such as TNF‐α, IL1, and IL6 in the localized area of bone defects promotes the production of osteoclasts.^[^ ^83^, ^84^ ^]^ Some studies have demonstrated that ROS plays an active role in osteoclast differentiation by activating the NF‐κB signaling pathway. Upon activation, the NF‐κB pathway increases the expression of *Nfatc1* and *Fos*, crucial transcription factors promoting osteoclast differentiation. Consistently, both ROS and NF‐κB signaling pathways were downregulated in the GH‐MCD group, and the ability of the GH‐MCD to inhibit the NF‐κB signaling pathway was stronger than that of GH‐M (Figure S5I, Supporting Information), suggesting that the GH‐MCD hydrogels suppressed inflammatory responses and osteoclast differentiation. Additionally, the GH‐MCD hydrogels increased the expression of *Spp1*, which promoted the osteogenic differentiation of BMSCs.^[^ ^47^ ^]^ Our results also suggest that the GH‐MCD hydrogel promotes osteogenic differentiation by modulating *Spp1* to promote the activation of β‐catenin and RUNX2. Altogether, the GH‐MCD hydrogels regulated the balance between bone formation and resorption in infectious bone defects by inhibiting osteoclast differentiation and promoting osteoblast differentiation, consequently promoting bone repair.

Furthermore, RNA‐seq showed that the GH‐MCD hydrogels decreased the activity of *Staphylococcus aureus* infection‐related pathways, highlighting their potent bactericidal activity. BMSCs treated with the GH‐MCD hydrogels had significantly lower expression of bacterial receptors and major complement genes, alleviating infection, attenuating inflammatory responses, and enhancing cellular defense against bacteria. In addition, the GH‐MCD hydrogels inhibited the mTOR pathway, which is negatively associated with autophagy and suppresses the formation of autophagosomes in cells.^[^ ^56^, ^85^, ^86^ ^]^ Autophagosomes are essential in clearing intracellular bacteria.^[^ ^57^, ^87^ ^]^ GH‐MCD hydrogels significantly enhanced autophagy in BMSCs. Additionally, the number of intracellular bacteria was reduced when co‐cultured with the GH‐MCD hydrogels, suggesting that the hydrogels promoted the production of autophagosomes by downregulating the mTOR signaling pathway and achieved sustained elimination of bacteria for long‐term bacteriostasis. Furthermore, CAMP, a cationic antimicrobial peptide important for cellular defense against bacterial infection,^[^ ^88^, ^89^ ^]^ was upregulated in BMSCs treated with the GH‐MCD hydrogels. CAMP synergizes with autophagosomes to maintain a healthy intracellular microenvironment and create favorable conditions for bone repair.

Notably, RNA‐seq showed that the GH‐MCD hydrogels played an important role in immune regulation by downregulating genes involved in hematopoietic cell lineage‐related pathways, complement and coagulation cascades, platelet activation, natural killer cell‐mediated cytotoxicity, B‐cell receptor signaling pathway, and osteoclast differentiation, suggesting that MCDs could reduce their immunogenicity. When a biomaterial is implanted into a host, a foreign body response (FBR) is triggered, wherein the host immune system recognizes the biomaterial as foreign, initiating a complex cascade of events mediated by various components,^[^ ^2^, ^12^, ^90^ ^]^ resulting in the fibrous encapsulation (leading to implantation failure) and unwanted degradation of the biomaterial. FBR can cause inflammation and tissue damage, leading to impaired bone healing. Moreover, implanted biomaterials are susceptible to bacterial infection. Bacterial toxins are potent activators of the host immune system and may lead to severe diseases.^[^ ^21^ ^]^ The MCDs developed in this study showed excellent performance in modulating the host immune response and minimizing FBR. However, further investigation is warranted to elucidate the underlying mechanisms.

In summary, the MCDs kill bacteria through the charge effect, inducing bacterial oxidative stress, and inhibiting bacterial growth and metabolism, meanwhile exerting antioxidant properties and providing a favorable microenvironment for cell growth and maintenance of intracellular ROS balance in the early stages. In bone formation, the MCDs kill intracellular bacteria by enhancing autophagy to prevent the recurrence of infection. At the same time, they regulate the wnt/β‐catenin signaling pathway by increasing the expression of *Spp1* to promote osteogenesis and inhibit osteoclast differentiation, thereby promoting the repair of infected bone defects. Although the multifunctional MCDs can program the transformation of the regenerative microenvironment and repair the infectious bone defect, further exploration of the mechanism is still needed.

## 4 Conclusion

In conclusion, we developed multifunctional MCDs with antibacterial and osteogenesis properties that can modulate the microenvironment of infectious bone defects. The hydrogels containing the MCDs released a large amount of MCDs in the early stage and maintained this release in the late stage in response to the acidic microenvironment of infectious bone defects. Upon release, the MCDs improved the ROS microenvironment, promoted osteogenesis, inhibited osteoclastogenesis, and exerted multimodal antimicrobial effects, eventually facilitating bone repair.

## 5 Experimental Section

### Preparation of the MCDs

Briefly, 0.2 g of melatonin was added to 10 mL of deionized water and dispersed evenly by sonication. Subsequently, the mixture was added into a 20 mL hydrothermal synthesis reaction kettle, heated by a muffle furnace at 180 °C for 4 h, filtered with 0.22 µm filter head after liquid cooling, and then dialyzed for 24 h (molecular weight cutoff: 500 Da).

### Characterizations of the MCDs

The morphology of MCDs was characterized using a transmission electron microscope (TEM, FEI, USA). The diameter distribution of MCD was measured using a nanoparticle size and zeta potential analyzer (Zetasizer Nano ZS90). The molecular structure of MCDs was detected by XPS (PHI 5000 Versaprobe III), FTIR (Nicolet 6700, Thermo Fisher Scientific, USA), XRD (D8 ADVANCE), and PL (Perkin Elmer LS‐55).

### Preparation of the GH‐MCD Composite Hydrogels

To synthesize HA‐CHO, 1.5 g of HA was dissolved in 150 mL of deionized water. Then 802 mg of sodium periodate was added and stirred for 2 h. The reaction was stopped by adding ethylene glycol (200 µL) and dialyzed against deionized water. The obtained HA‐CHO was freeze‐dried and stored at 4 °C.

To obtain GH hydrogel, 1 g of GelMA sponge, 50 mg of photoinitiator lithium phenyl‐2,4,6‐trimethyl‐benzoyl phosphinate, and 100 mg of HA‐CHO were dissolved in 20 mL of deionized water at 37 °C, and then photocrosslinked by blue light (405 nm) for 1 min. To prepare the GH‐MCD composite hydrogels containing different amounts of MCDs, 125, 250 and 500 µg MCD were added into 1 mL GH hydrogel precursor solution to form the composite hydrogels of GH‐125 MCD, GH‐250 MCD, GH‐500 MCD after photocrosslinking.

### Characterization of the Morphology of GH‐MCD Composite Hydrogels

The GH hydrogels and GH‐MCD hydrogels were immersed in PBS solution of pH 7.2 and pH 5.5, and then incubated in an incubator at 37 °C for 24 h. Subsequently, freeze‐drying was carried out in a lyophilizer, and the lyophilized samples were cut into thin slices (the thickness of the slices was ≈2 mm) and observed by SEM.

### Mechanical Tests of the Hydrogels

Ninety microliters of GH, GH‐125 MCD, GH‐250 MCD, and GH‐500 MCD composite hydrogels were sucked into a decapitated 1 mL syringe, and then photo crosslinked for 1 min using a blue light (405 nm), and then a cylindrical composite hydrogel sample with a diameter of 5 mm and a height of 5 mm was obtained. The sample was placed on the platform of the universal mechanical testing machine, and the height of the compression platform was adjusted to be in contact with the composite hydrogel, then, all the parameters were cleared to zero, and the compression speed was set to 5 mm min^−1^. The test ended when the mechanical curve showed a decreasing trend.

### Release of MCDs from the Hydrogels

The GH‐500 MCD composite hydrogel was prepared, immersed into a buffer with pH 7.2 and pH 5.5, and then incubated in an oven at 37 °C. The buffer was incubated at 37 °C in an oven, and the buffer was aspirated at 1, 2, 3, 4, 5, 6, 7, 9, 11, 14, and 21 days, and 5 mL of fresh buffer was supplemented at the same time. Finally, the released amount of MCDs was detected by UV–vis spectrophotometer at 305 nm.

### Biocompatibility Test of the Composite Hydrogels

BMSCs were co‐cultured with hydrogels for 1 day, 3 days, and 5 days, and then the proliferation of cells was detected by CCK‐8 assay (Dojindo, Japan). After 3 days, the effect of GH‐MCD composite hydrogel on cell adhesion was observed by cytoskeleton staining.

### DPPH Experiment

DPPH radical was a stable free radical with a purple color in its alcohol solution and strong absorption at 515 nm. Antioxidants scavenge DPPH free radicals, making the purple color lighter and the absorbance decrease. The decrease in absorbance was proportional to the ability of the sample to scavenge DPPH radicals within a certain range. The hydrogels of each group were immersed in the working solution and reacted to avoid light for half an hour (the rest of the groups were set according to the instructions), and the absorbance was detected at the wavelength of 515 nm, and the clearance rate of DPPH radicals was calculated according to the formula in the kit.

### Intracellular ROS Detection

BMSCs were seeded in 12‐well plates (3 × 10^4^ cells per well). After the cells were attached to the well plates, the medium was replaced with α‐MEM complete medium containing H_2_O_2_ (100 µm) for 12 h and then replaced with a new α‐MEM complete medium. Then the hydrogels were co‐cultured with BMSC for 12 h. The internal ROS of BMSCs was stained by DCFH‐DA and photographed using an inverted fluorescence microscope.

### Intracellular Antioxidant Enzyme Detection

Cells were treated as above, followed by the extraction of total protein from BMSCs using Ripa, and the SOD and CAT enzyme activities were detected according to the methods shown in the SOD and CAT kits (Beyotime).

### Antioxidant Genes and Proteins

Cells were treated as above. Antioxidant gene transcription (*Nrf2*, *Sirt1*) and protein expression (SOD, CAT) levels were detected using real‐time fluorescence quantitative PCR and Western blot (gene sequences and antibody information are provided in Table S1, Supporting Information).

### Bacterial Coating Experiment

Briefly, 20 µL of *S. aureus* suspension at a concentration of 10^6^ CFU mL^−1^ was added to the surface of each group of hydrogels, and *S. aureus* was resuspended with PBS solution for 24 h, after which, an appropriate amount of *S. aureus* suspension was taken and evenly coated on the surface of LB agar plates, and continued to incubate for 18–24 h. The colonies on the surface of LB agar plates were counted and analyzed using Image J.

### AO/EB Staining

An appropriate amount of *S. aureus* suspension (20 µL *S. aureus* suspension per well) was added onto the surface of different hydrogels, and then 1 mL LB medium was added to the well. The growth of *S. aureus* on the surface of hydrogels was observed by AO/EB staining after 24 h of incubation.

### Antimicrobial Kinetics

Appropriate amount of *S. aureus* suspension (20 µL *S. aureus* suspension per hydrogel) was added to the surface of each group of hydrogels, and the *S. aureus* was resuspended using PBS at 0, 2, 4, 8, 12, and 24 h respectively. Then, the appropriate amount of *S. aureus* suspension was taken and evenly coated on the surface of LB agar plates.

### The Morphology of Bacteria on the Hydrogels

An appropriate amount of *S. aureus* suspension (20 µL *S. aureus* suspension per hydrogel) was added to the surface of hydrogels, and the morphology of the bacteria was observed by SEM after 24 h of incubation.

### Detection of ROS in Bacteria

*S. aureus* was seeded on the surface of each hydrogel at a density of 1 × 10^8^ CFU mL^−1^ per well. Then, the bacterial suspension was aspirated after 30 min of incubation, centrifuged, and then rinsed with PBS. After that, DCFH‐DA was added, and the level of ROS was detected by fluorescence microplate (excitation wavelength of 488 nm).

### Bacterial Biofilm Staining

The formation of bacterial biofilm was observed by crystal violet staining after 48 h of co‐culturing each group of hydrogels with bacteria. After decolorization with glacial acetic acid, the absorbance was measured at 595nm.

### ONPG Assay

After co‐culturing the bacteria with different hydrogels for 1 day, the bacterial membrane permeability was detected by ONPG assay, and the absorbance was measured at 405 nm.

### The secA2 Expression of Bacteria

After co‐culturing the bacteria with different hydrogels for 1 day, the *secA2* gene of *S. aureus* was detected by qPCR. The groups were as follows: The groups were as follows: GH group (bacteria cultured with GH hydrogel), GH‐MCD group (bacteria cultured with GH‐500 MCD composite hydrogel).

### In Vitro Osteogenesis Properties

BMSCs were seeded into 12‐well plates (3 × 10^4^ cells per well), and the medium was changed to osteogenic differentiation medium (10 mm sodium β‐glycerophosphate, 10 mm dexamethasone, and 50 µg mL^−1^ ascorbic acid, α‐MEM complete medium) after 24 h and co‐cultivated with different hydrogels. The medium was changed every two days. After 7 days, ALP staining was performed, and ALP activity was quantified using the ALP assay kit. After 14 days, ARS was performed, followed by the dissolution of calcium nodules with perchloric acid to make a quantitative analysis by measuring the absorbance at 420 nm. Osteogenic gene transcription levels (*Alpl*, *Runx2*, *Cola1*, *Bglap*) and protein expression (ALP, RUNX2, COL1, OCN) levels were detected by qPCR and Western blot at 7 and 14 days of osteogenic induction, respectively (gene sequences and antibody information are provided in Supporting Information). The groups were as follows: Ctrl group (cultured in α‐MEM medium), Induced group (cultured in osteoinductive medium), GH group (cultured in osteoinductive medium plus GH hydrogel), GH‐250 MCD group (cultured in osteoinductive medium plus GH‐250 MCD composite hydrogel), and GH‐500 MCD group (cultured in osteoinductive medium plus GH‐500 MCD composite hydrogel).

### In Vivo ROS Detection

C57 mice were randomly divided into 3 groups: *S. aureus* group (only *S. aureus* was added), GH group (GH hydrogel + *S. aureus*), and GH‐MCD group (GH‐500 MCD composite hydrogel + *S. aureus*). The mice were anesthetized by intraperitoneal injection of sodium pentobarbital, then shaved and disinfected. A drill was used to form a 2.5 mm diameter circular full‐layer bone defect in the cranium of the mice, which was then filled with different types of hydrogels and irradiated using blue light with a wavelength of 405 nm for 1 min, followed by the addition of 10 µL of 10^4^ CFU mL^−1^ of *S. aureus* to the injury site. ROS Brite 700 was injected into the cranial region of mice at 3 and 7 days after surgery, and fluorescent images were acquired using an in vivo imaging system (PerkinElmer, Boston, USA). Fluorescent images were recorded to assess ROS production in the cranial defect area of the mice.

### In Vivo Antibacterial and Bone Repair Experiments

A model of infected bone defects in femoral condyles was established using Sprague–Dawley rats, which were shaved and sterilized after intraperitoneal anesthesia. The bone defect with a diameter of 3 mm and a depth of 3 mm was formed with a drill, with the left and right sides modeled. Then, it was filled with different kinds of hydrogels. After which, 10 µL of 10^7^ CFU mL^−1^ of *S. aureus* was added to the defect site. In the control group, only *S. aureus* was added to the defect site without hydrogel filling. For the in vivo antibacterial experiment, the rats were sacrificed at 3 and 14 days after surgery, and femur specimens were collected and immersed in PBS, and a certain amount of PBS was taken for the bacterial coating experiment. For the in vivo bone repair experiment, rats were sacrificed at 4 and 8 weeks after surgery, and then femur specimens were collected for subsequent experiments. The groups were as follows. In vivo antibacterial experiment: *S. aureus* group (only *S. aureus* added), GH group (GH hydrogel + *S. aureus*), GH‐MCD group (GH‐500 MCD composite hydrogel + *S. aureus*). In vivo bone repair experiments: Ctrl group (pure defect group, without S. aureus), S. aureus group (only *S. aureus* added), GH group (GH hydrogel + *S. aureus*), GH‐MCD group (GH‐500 MCD composite hydrogel + *S. aureus*).

### Micro‐CT Scanning and Analysis

Femoral samples were soaked in formalin for 12 h, then rinsed with PBS and scanned and analyzed using Micro‐CT (NEMO Micro CT). Quantitative analysis of bone volume fraction, trabecular thickness, number, and trabecular separation at femoral defects was performed using AVATAR3 software.

### H&E Staining

The femur samples were decalcified using 14% Ethylene diamine tetraacetic acid (EDTA) solution, and the EDTA solution was changed once a day for ≈1 month. Then, the decalcified femur samples were dehydrated and paraffin‐embedded. The effect of the composite hydrogel on the repair of infected bone defects was observed by H&E staining.

### In Vivo Biosafety and Stability of GH‐MCD Hydrogels

A model of subcutaneous embedded hydrogel was used in C57 mice which were shaved and sterilized after intraperitoneal anesthesia. A 5 mm long incision was made on the back of the mouse and it was filled with GH‐MCD hydrogels. Heart, liver, spleen, lung, and kidney samples were collected at 7 and 14 days and stained with H&E staining. The groups were as follows: Ctrl group and GH‐MCD group (filled with GH‐500 MCD composite hydrogel).

### RNA Sequencing

BMSCs were seeded into 6‐well plates (2 × 10^5^ cells per well), and the medium was changed to osteogenic differentiation medium after 24 h and co‐cultivated with different hydrogels. The medium was changed every two days. After 5 days, samples were collected for RNA sequencing analysis.

### siRNA Interference

BMSCs were plated on 6 well culture plates 1 day before transfection. siRNA transfections were performed with Lipofectamine 2000 (Thermo Fisher Scientific) in antibiotic‐free and FBS‐free medium according to the manufacturer's instructions. The siRNA sequences were as follows: sense strand 5′‐GGAUGAACCAAGCGUGGAATT‐3′ and anti‐sense strand 5′‐UUCCACGCUUGGUUCAUCCTT‐3′. The negative control sequences were as follows: sense strand 5′‐UUCUCCGAACGUGUCACGUTT‐3′ and anti‐sense strand 5′‐ACGUGACACGUU CGG AGA ATT‐3′. After 24 h, the cells were further treated with GH‐MCD. Then, osteogenic gene transcription levels (*Alpl*, *Runx2*) and protein expression (β‐catenin, RUNX2) levels were detected by qPCR and Western blot at 5 days of osteogenic induction, respectively.

### TRAP Staining and Podosome Belt Formation Assay

BMM was seeded into 24‐well plates (1×10^5^ cells per well), and after 24 h, the prepared hydrogels were added to the cell culture plates, and the medium in the cell culture plates was replaced with osteoblast‐inducing medium, and the medium was changed every other day, and the incubation was continued for 7 days to detect the formation of osteoblasts by TRAP staining kit, and the formation of Podosome belt was detected by rhodamine‐labeled phalloidin staining. Osteoclasts were defined as cells with nuclei greater than 3. After 7 days, protein and RNA were collected for real‐time fluorescence quantitative PCR and Western blot to detect the expression of osteoclast‐related proteins and genes.

### Autophagy‐Related Experiments

BMSC were seeded in 6‐well plates (2 × 10^5^ cells per well) for 24 h, the prepared hydrogels were added to the cell culture plates, and the α‐MEM complete medium was changed every other day. After 5 days of culture, the expression of autophagy‐related proteins was detected by immunofluorescence and Western blot.

### Fluorescence‐Labeled Cells and Bacteria in Co‐Culture

BMSCs and *S. aureus* (BMSCs were labeled with DiO with green fluorescence and *S. aureus* were labeled with DiD with red fluorescence) were seeded simultaneously on the surface of the hydrogel, and appropriate culture medium was added (the medium consisted of 80% α‐MEM complete medium and 20% LB medium), and then cultured for 24 h. After 24 h, the extracellular bacteria were eliminated by gentamicin (60 µg mL^−1^). The growth of intracellular bacteria was observed with an inverted fluorescence microscope.

### Bacterial Coating Experiment

Briefly, 20 µL of *E. coli* suspension at a concentration of 10^6^ CFU mL^−1^ was added to the surface of each group of hydrogels, and *E. coli* was resuspended with PBS solution for 24 h, after which, an appropriate amount of *E. coli* suspension was taken and evenly coated on the surface of LB agar plates, and continued to incubate for 18 to 24 h.

### The Morphology of Bacteria on the Hydrogels

An appropriate amount of *E. coli* suspension (20 µL *E. coli* suspension per hydrogel) was added to the surface of the hydrogels, and the morphology of the bacteria was observed by SEM after 24 h of incubation.

### Statistical Analysis

Each experimental group had 3 or more replicates, and all data were expressed as “mean ± standard deviation”, and the data were analyzed by GraphPad Prism 8.0 and Origin Pro 2021, and compared with each other using the One‐way ANOVA test. The difference between the two groups was considered statistically significant when the *p*‐value was less than 0.05.

## Conflict of Interest

The authors declare no conflict of interest.

## Supporting information



Supporting Information

## Data Availability

The data that support the findings of this study are available from the corresponding author upon reasonable request.

## References

[1] N. Kavanagh , E. J. Ryan , A. Widaa , G. Sexton , J. Fennell , S. O'Rourke , K. C. Cahill , C. J. Kearney , F. J. O'Brien , S. W. Kerrigan , Clin. Microbiol. Rev. 2018, 31, e00084. 10.1128/CMR.00084-17 PMC5967688 29444953

[2] J. Lei , C. F. Wang , X. B. Feng , L. Ma , X. M. Liu , Y. Luo , L. Tan , S. L. Wu , C. Yang , Chem. Eng. J. 2022, 435, 134624.

[3] C. R. Arciola , D. Campoccia , L. Montanaro , Nat. Rev. Microbiol. 2018, 16, 397. 29720707 10.1038/s41579-018-0019-y

[4] E. E. Huang , N. Zhang , E. A. Ganio , H. Shen , X. Li , M. Ueno , T. Utsunomiya , M. Maruyama , Q. Gao , N. Su , Z. Yao , F. Yang , B. Gaudillière , S. B. Goodman , J. Orthop. Transl. 2022, 36, 64. 10.1016/j.jot.2022.05.010 PMC9357712 35979174

[5] M. Wang , H. Li , Y. Yang , K. Yuan , F. Zhou , H. Liu , Q. Zhou , S. Yang , T. Tang , Bioact. 2021, 6, 1318. 10.1016/j.bioactmat.2020.10.022 PMC7658329 33210025

[6] O. C. Koldsland , A. A. Scheie , A. M. Aass , J. Periodontol. 2009, 80, 1069. 19563286 10.1902/jop.2009.080594

[7] C. H. Mi , X. Y. Qi , Y. W. Ding , J. Zhou , J. W. Dao , D. X. Wei , Biomater. Transl. 2023, 4, 234. 38282701 10.12336/biomatertransl.2023.04.004 PMC10817797

[8] K. A. Tucker , S. S. Reilly , C. S. Leslie , M. C. Hudson , FEMS Microbiol. Lett. 2000, 186, 151. 10802163 10.1111/j.1574-6968.2000.tb09096.x

[9] I. Marriott , Immunol. Res. 2004, 30, 291. 15531771 10.1385/IR:30:3:291

[10] G. B. Schneider , R. Zaharias , D. Seabold , C. Stanford , J. Orth. Res. 2011, 29, 1443. 10.1002/jor.21382 21412826

[11] W. Zhou , X. Peng , Y. Ma , Y. Hu , Y. Wu , F. Lan , M. D. Weir , M. Y. Li , B. Ren , T. W. Oates , H. H. K. Xu , X. D. Zhou , L. Cheng , Acta Biomater. 2020, 101, 128. 31629895 10.1016/j.actbio.2019.10.023

[12] L. Qin , S. Yang , C. Zhao , J. Yang , F. Li , Z. Xu , Y. Yang , H. Zhou , K. Li , C. Xiong , W. Huang , N. Hu , X. Hu , Bone Res. 2024, 12, 28. 38744863 10.1038/s41413-024-00332-w PMC11094017

[13] J. Shan , X. Wu , J. Che , J. Gan , Y. Zhao , Adv. Sci. 2024, 11, 2309622. 10.1002/advs.202309622 PMC11186059 38582511

[14] L. Foulston , K. W. Elsholz Alexander , S. DeFrancesco Alicia , R. Losick , MBio. 2014, 5, 01667. 10.1128/mBio.01667-14 PMC4173787 25182325

[15] W. H. Bowen , R. A. Burne , H. Wu , H. Koo , Trends Microbiol. 2018, 26, 229. 29097091 10.1016/j.tim.2017.09.008 PMC5834367

[16] Y. Liu , X. H. Zhang , C. Cao , Y. L. Zhang , J. Q. Wei , Y. J. Li , W. W. Liang , Z. W. Hu , J. X. Zhang , Y. Wei , X. L. Deng , Adv. Funct. Mater. 2017, 27, 1703771.

[17] M. Caldwell , M. Hughes , F. Wei , C. Ngo , R. Pascua , A. S. Pugazhendhi , M. J. Coathup , Bone Res. 2023, 11, 14. 36894568 10.1038/s41413-023-00254-z PMC9998894

[18] Q. Gao , Z. Chen , X. Yang , Macromol. Rapid Commun. 2023, 44, 2300453. 10.1002/marc.202300453 37800610

[19] Y. Deng , X. Y. Shi , Y. Chen , W. Z. Yang , Y. Ma , X. L. Shi , P. G. Song , M. S. Dargusch , Z. G. Chen , Ind. Eng. Chem. Res. 2020, 59, 12123.

[20] C. Sun , Z. Y. Wang , L. D. Yue , Q. X. Huang , S. Y. Lu , R. B. Wang , J. Mater. Chem. B. 2020, 8, 8878. 33026388 10.1039/d0tb01475c

[21] T. Finkel , J. Cell Biol. 2011, 194, 7. 21746850 10.1083/jcb.201102095 PMC3135394

[22] Y. F. Feng , L. Wang , Y. Zhang , X. Li , Z. S. Ma , J. W. Zou , W. Lei , Z. Y. Zhang , Biomaterials. 2013, 34, 2234. 23294547 10.1016/j.biomaterials.2012.12.023

[23] Q. Huang , B. Gao , Q. Jie , B.‐Y. Wei , J. Fan , H. Y. Zhang , J. K. Zhang , X. J. Li , J. Shi , Z. J. Luo , L. Yang , J. Liu , Bone. 2014, 66, 306. 24933344 10.1016/j.bone.2014.06.010

[24] X. Li , Y. Kou , J. Jia , M. Liu , R. Gao , Y. Li , G. Li , S. Xu , W. Song , Y. Xie , X. Li , T. Zhao , Nano Res. 2024, 17, 9898.

[25] Y. Xu , Y. Luo , Z. Weng , H. Xu , W. Zhang , Q. Li , H. Liu , L. Liu , Y. Wang , X. Liu , L. Liao , X. Wang , ACS Nano. 2023, 17, 18732. 37768714 10.1021/acsnano.3c01940

[26] L. Ðorđević , F. Arcudi , M. Cacioppo , M. Prato , Nat. Nanotechnol. 2022, 17, 112. 35173327 10.1038/s41565-021-01051-7

[27] H. Su , W. Wang , R. Shi , H. Tang , L. Sun , L. Wang , Q. Liu , T. Zhang , Carbon Energy. 2023, 5, e280.

[28] Y. Liu , J. H. Lei , G. Wang , Z. Zhang , J. Wu , B. Zhang , H. Zhang , E. Liu , L. Wang , T.‐M. Liu , G. Xing , D. Ouyang , C. X. Deng , Z. Tang , S. Qu , Adv. Sci. 2022, 9, 2202283.

[29] L. Jiang , H. Cai , W. Zhou , Z. Li , L. Zhang , H. Bi , Adv. Mater. 2023, 35, 2210776. 10.1002/adma.202210776 36645339

[30] H. Zhao , X. Jia , M. Zhang , L. Zhu , Macromol. Rapid Commun. 2024, 45, 2300538. 10.1002/marc.202300538 37877956

[31] J. Liu , T. Kong , H. M. Xiong , Adv. Mater. 2022, 34, 2200152. 10.1002/adma.202200152 35229375

[32] Y. Wang , X. Li , S. Zhao , B. Wang , X. Song , J. Xiao , M. Lan , Coord. Chem. Rev. 2022,470, 214703.

[33] L. Yao , M. M. Zhao , Q. W. Luo , Y. C. Zhang , T. T. Liu , Z. Yang , M. Liao , P. Tu , K. W. Zeng , ACS Nano. 2022, 16, 9228. 35622408 10.1021/acsnano.2c01619

[34] P. Muhammad , S. Hanif , J. Li , A. Guller , F. U. Rehman , M. Ismail , D. Zhang , X. Yan , K. Fan , B. Shi , Nano Today. 2022, 45, 101530.

[35] C. Liu , W. Fan , W. X. Cheng , Y. Gu , Y. Chen , W. Zhou , X. F. Yu , M. Chen , M. Zhu , K. Fan , Q. Y. Luo , Adv. Funct. Mater. 2023, 33, 2213856.

[36] Q. Ci , Y. Wang , B. Wu , E. Coy , J. Li , D. Jiang , P. Zhang , G. Wang , Adv. Sci. 2023, 10, 2206271. 10.1002/advs.202206271 PMC9982550 36596672

[37] Y. Liu , Y. Li , S. Koo , Y. Sun , Y. Liu , X. Liu , Y. Pan , Z. Zhang , M. Du , S. Lu , X. Qiao , J. Gao , X. Wang , Z. Deng , X. Meng , Y. Xiao , J. S. Kim , X. Hong , Chem. Rev. 2022, 122, 209. 34664951 10.1021/acs.chemrev.1c00553

[38] B. Geng , P. Li , F. Fang , W. Shi , J. Glowacki , D. Pan , L. Shen , Carbon. 2021, 184, 375.

[39] Y. Cui , H. Liu , Y. Tian , Y. Fan , S. Li , G. Wang , Y. Wang , C. Peng , D. Wu , Mater. Today Bio. 2022, 16, 100409. 10.1016/j.mtbio.2022.100409 PMC9449864 36090611

[40] X. Hao , L. Huang , C. Zhao , S. Chen , W. Lin , Y. Lin , L. Zhang , A. a. Sun , C. Miao , X. Lin , M. Chen , S. Weng , Mater. Sci. Eng. C. 2021, 123, 111971. 10.1016/j.msec.2021.111971 33812599

[41] W. B. Zhao , K. K. Liu , Y. Wang , F. K. Li , R. Guo , S. Y. Song , C. X. Shan , Adv. Healthcare Mater. 2023, 12, 2300324. 10.1002/adhm.202300324 37178318

[42] A. Verma , F. Arshad , K. Ahmad , U. Goswami , S. K. Samanta , A. K. Sahoo , M. P. Sk , Nanotechnology. 2020, 31, 095101. 31703210 10.1088/1361-6528/ab55b8

[43] J. Wang , Y. Fu , Z. Gu , H. Pan , P. Zhou , Q. Gan , Y. Yuan , C. Liu , Small. 2023, 20, 2303773. 10.1002/smll.202303773 37702145

[44] H. Wang , Z. Song , J. Gu , S. Li , Y. Wu , H. Han , ACS Biomater. Sci. Eng. 2019, 5, 4739. 33448817 10.1021/acsbiomaterials.9b00583

[45] H. J. Jian , R.‐S. Wu , T. Y. Lin , Y. J. Li , H. J. Lin , S. G. Harroun , J. Y. Lai , C. C. Huang , ACS Nano. 2017, 11, 6703. 28677399 10.1021/acsnano.7b01023

[46] P. Li , S. Liu , X. Yang , S. Du , W. Tang , W. Cao , J. Zhou , X. Gong , X. Xing , Chem. Eng. J. 2021, 403, 126387.

[47] J. Li , J. Ma , H. Sun , M. Yu , H. Wang , Q. Meng , Z. Li , D. Liu , J. Bai , G. Liu , X. Xing , F. Han , B. Li , Sci. Adv. 2023, 9, eadf8645. 37235658 10.1126/sciadv.adf8645 PMC10219602

[48] C. Ren , X. Hao , L. Wang , Y. Hu , L. Meng , S. Zheng , F. Ren , W. Bu , H. Wang , D. Li , K. Zhang , H. Sun , Adv. Healthcare Mater. 2021, 10, e2100196. 10.1002/adhm.202100196 33987977

[49] K. K. Monteiro , M. E. Shiroma , L. L. Damous , M. D. Simões , R. D. Simões , J. Cipolla‐Neto , E. C. Baracat , J. M. Soares‐Jr , Antioxidants. 2024, 13, 13040439. 10.3390/antiox13040439 PMC11047453 38671887

[50] D. P. Cardinali , J. Pineal Res. 2024, 76, e12931. 38083808 10.1111/jpi.12931

[51] F. He , X. Wu , Q. Zhang , Y. Li , Y. Ye , P. Li , S. Chen , Y. Peng , R. Hardeland , Y. Xia , Front. Immunol. 2021, 12, 683879. 34135911 10.3389/fimmu.2021.683879 PMC8201398

[52] Y. Miao , Y. Chen , X. Liu , J. Diao , N. Zhao , X. Shi , Y. Wang , J. Mater. Chem. B. 2019, 7, 3250.

[53] S. Maria , M. H. Swanson , L. T. Enderby , F. D'Amico , B. Enderby , R. M. Samsonraj , A. Dudakovic , A. J. van Wijnen , P. A. Witt‐Enderby , Aging. 2017, 9, 256. 28130552 10.18632/aging.101158 PMC5310667

[54] W. Song , Z. Ma , C. Wang , H. Li , Y. He , J. Mater. Chem. B. 2019, 7, 6564. 31588948 10.1039/c9tb01516g

[55] X. Xin , J. Liu , X. Liu , Y. Xin , Y. Hou , X. Xiang , Y. Deng , B. Yang , W. Yu , ACS Nano. 2024, 18, 8307. 38437643 10.1021/acsnano.3c12580

[56] Z. Wang , R. Lan , Y. Xu , J. Zuo , X. Han , V. Phouthapane , Z. Luo , J. Miao , Front. Immunol. 2021, 12, 631113. 33777017 10.3389/fimmu.2021.631113 PMC7996097

[57] M. Xu , X. Z. Zhong , P. Huang , D. Jaślan , P. Wang , X. Sun , E.‐M. Weiden , Y. El Hiani , C. Grimm , X.‐P. Dong , Proc. Natl. Acad. Sci. USA. 2023, 120, e2215777120. 37585464 10.1073/pnas.2215777120 PMC10450854

[58] C. Yin , Q. Zhao , W. Li , Z. Zhao , J. Wang , T. Deng , P. Zhang , K. Shen , Z. Li , Y. Zhang , Acta Biomater. 2020, 102, 416. 31760223 10.1016/j.actbio.2019.11.025

[59] J. Xu , Y. Wang , J. Li , X. Zhang , Y. Geng , Y. Huang , K. Dai , X. Zhang , Cell Death Differ. 2016, 23, 1941. 27472064 10.1038/cdd.2016.72 PMC5136484

[60] J. E. Janis , B. Harrison , Plast. Reconstr. Surg. 2014, 133, 199E. 10.1097/01.prs.0000437224.02985.f9 24469191

[61] J. Muri , M. Kopf , Nat. Rev. Immunol. 2021, 21, 363. 33340021 10.1038/s41577-020-00478-8

[62] H. Wei , H. Huang , H. He , Y. Xiao , L. Chun , Z. Jin , H. Li , L. Zheng , J. Zhao , Z. Qin , Research. 2024, 7, 0310. 38410279 10.34133/research.0310 PMC10895487

[63] B. Schumacher , J. Pothof , J. Vijg , J. H. J. Hoeijmakers , Nature. 2021, 592, 695. 33911272 10.1038/s41586-021-03307-7 PMC9844150

[64] C. Matyas , G. Haskó , L. Liaudet , E. Trojnar , P. Pacher , Nat. Rev. Cardiol. 2021, 18, 117. 32999450 10.1038/s41569-020-0433-5

[65] L. Chen , J. Yang , Z. Cai , Y. Huang , P. Xiao , H. Chen , X. Luo , W. Huang , W. Cui , N. Hu , Research. 2024, 7, 0306. 38274127 10.34133/research.0306 PMC10809599

[66] Y. Zhang , Z. Zou , S. Liu , F. Chen , M. Li , H. Zou , H. Liu , J. Ding , Asian J Pharm. Sci. 2024, 19, 10088. 10.1016/j.ajps.2024.100886 PMC10999513 38590795

[67] E. Giusto , G. Blunn , R. F. de Godoy , C. Liu , C. Pendegrass , Biomater. Transl. 2022, 3, 243. 36846509 10.12336/biomatertransl.2022.04.004 PMC9947732

[68] G. Walters , I. Pountos , P. V. Giannoudis , J. Tissue Eng. Regener. Med. 2018, 12, e1662. 10.1002/term.2593 29047220

[69] N. K. Lee , Endocrinol. Metab. 2010, 25, 264.

[70] A. Brandao‐Burch , J. C. Utting , I. R. Orriss , T. R. Arnett , Calcif. Tissue Int. 2005, 77, 167. 16075362 10.1007/s00223-004-0285-8

[71] Y. H. Chan , K. N. Ho , Y. C. Lee , M.‐J. Chou , W. Z. Lew , H. M. Huang , P. C. Lai , S. W. Feng , Stem Cell. Res. Ther. 2022, 13, 73. 35183254 10.1186/s13287-022-02744-z PMC8858457

[72] X. Liu , Y. Gong , K. Xiong , Y. Ye , Y. Xiong , Z. Zhuang , Y. Luo , Q. Jiang , F. He , J. Pineal Res. 2013, 55, 14. 23488678 10.1111/jpi.12045

[73] O. F. Tekbas , R. Ogur , A. Korkmaz , A. Kilic , R. J. Reiter , J. Pineal Res. 2008, 44, 222. 18289175 10.1111/j.1600-079X.2007.00516.x

[74] B. Sun , F. Wu , Q. Zhang , X. Chu , Z. Wang , X. Huang , J. Li , C. Yao , N. Zhou , J. Shen , J. Colloid Interface Sci. 2021, 584, 505. 33129160 10.1016/j.jcis.2020.10.015

[75] N. Wang , L. Feng , X. D. Xu , S. Feng , Macromol. Rapid Commun. 2022, 43, 2100885. 10.1002/marc.202100885 35112755

[76] T. Han , Y. Wang , S. Ma , M. Li , N. Zhu , S. Tao , J. Xu , B. Sun , Y. Jia , Y. Zhang , S. Zhu , B. Yang , Adv. Sci. 2022, 9, 2203474. 10.1002/advs.202203474 PMC9596834 36047633

[77] P. Gao , H. Hui , C. Guo , Y. Liu , Y. Su , X. Huang , K. Guo , W. Shang , J. Jiang , J. Tian , Carbon. 2023, 201, 805.

[78] Y. Wang , X. Hao , H. Peng , X. Zhou , X. Xie , Macromol. Rapid Commun. 2022, 43, 2100868. 10.1002/marc.202100868 35021265

[79] G. Jeong , T. Kim , S. D. Park , M. J. Yoo , C. H. Park , H. Yang , Macromol. Rapid Commun. 2024, 45, 2300542. 10.1002/marc.202300542 38014607

[80] S. Swanson , R. Ioerger Thomas , W. Rigel Nathan , K. Miller Brittany , M. Braunstein , C. Sacchettini James , J. Bacteriol. 2016, 198, 720. 10.1128/JB.00696-15 PMC4751807 26668263

[81] S. Ligon Lauren , W. Rigel Nathan , A. Romanchuk , D. Jones Corbin , M. Braunstein , J. Bacteriol. 2013, 195, 4456. 23913320 10.1128/JB.00630-13 PMC3807457

[82] A. Widaa , T. Claro , T. J. Foster , F. J. O'Brien , S. W. Kerrigan , PLoS One. 2012, 7, e40586. 22792377 10.1371/journal.pone.0040586 PMC3394727

[83] N. Somayaji Shankari , S. Ritchie , M. Sahraei , I. Marriott , C. Hudson Michael , Infect. Immun. 2008, 76, 5120. 18765718 10.1128/IAI.00228-08 PMC2573315

[84] O. Kudo , Y. Fujikawa , I. Itonaga , A. Sabokbar , T. Torisu , N. A. Athanasou , J. Pathol. 2002, 198, 220. 12237882 10.1002/path.1190

[85] J. Cosin‐Roger , S. Simmen , H. Melhem , K. Atrott , I. Frey‐Wagner , M. Hausmann , C. de Vallière , M. R. Spalinger , P. Spielmann , R. H. Wenger , J. Zeitz , S. R. Vavricka , G. Rogler , P. A. Ruiz , Nat. Commun. 2017, 8, 98. 28740109 10.1038/s41467-017-00213-3 PMC5524634

[86] F. Nazio , F. Strappazzon , M. Antonioli , P. Bielli , V. Cianfanelli , M. Bordi , C. Gretzmeier , J. Dengjel , M. Piacentini , G. M. Fimia , F. Cecconi , Nat. Cell Biol. 2013, 15, 406. 23524951 10.1038/ncb2708

[87] J. Huang , J. H. Brumell , Nat. Rev. Microbiol. 2014, 12, 101. 24384599 10.1038/nrmicro3160 PMC7097477

[88] N. Mookherjee , R. E. W. Hancock , Cell. Mol. Life Sci. 2007, 64, 922. 17310278 10.1007/s00018-007-6475-6 PMC11136131

[89] M. Magana , M. Pushpanathan , A. L. Santos , L. Leanse , M. Fernandez , A. Ioannidis , M. A. Giulianotti , Y. Apidianakis , S. Bradfute , A. L. Ferguson , A. Cherkasov , M. N. Seleem , C. Pinilla , C. de la Fuente‐Nunez , T. Lazaridis , T. Dai , R. A. Houghten , R. E. W. Hancock , G. P. Tegos , J. Infect. Dis. 2020, 20, e216. 10.1016/S1473-3099(20)30327-3 32653070

[90] S. B. Goodman , Z. Yao , M. Keeney , F. Yang , Biomaterials. 2013, 34, 3174. 23391496 10.1016/j.biomaterials.2013.01.074 PMC3582840

