# Supplementary material for: Programmed Transformation of Osteogenesis Microenvironment by a Multifunctional Hydrogel to Enhance Repair of Infectious Bone Defects
Source: Adv Sci (Weinh). 2025 Jan 22;12(10):2409683. doi: 10.1002/advs.202409683 (PMC11904992; doi:10.1002/advs.202409683)
Supplement: Supplementary file 1 — Supporting Information [file ADVS-12-2409683-s001.docx]

Supplementary Materials for

**Programmed transformation of osteogenesis microenvironment by a multifunctional hydrogel to enhance repair of infectious bone defects**

*En Xie^1^,* *Zhangqin Yuan^1^, Qianglong Chen^1^, Jie Hu, Jiaying Li, Kexin Li, Huan Wang,* *Jinjin Ma, Bin Meng, Ruoxi Zhang, Haijiao Mao, Ting Liang,* *Lijie Wang, Chaoyong Liu^*^,* *Bin Li^*^, Fengxuan Han^*^*

*Corresponding author Email: fxhan@suda.edu.cn, binli@suda.edu.cn, chaoyongliu@mail.buct.edu.cn.

**This file includes:**

Supplementary Text

Figs. S1 to S5

Tables S1

**Supplementary Text**

**
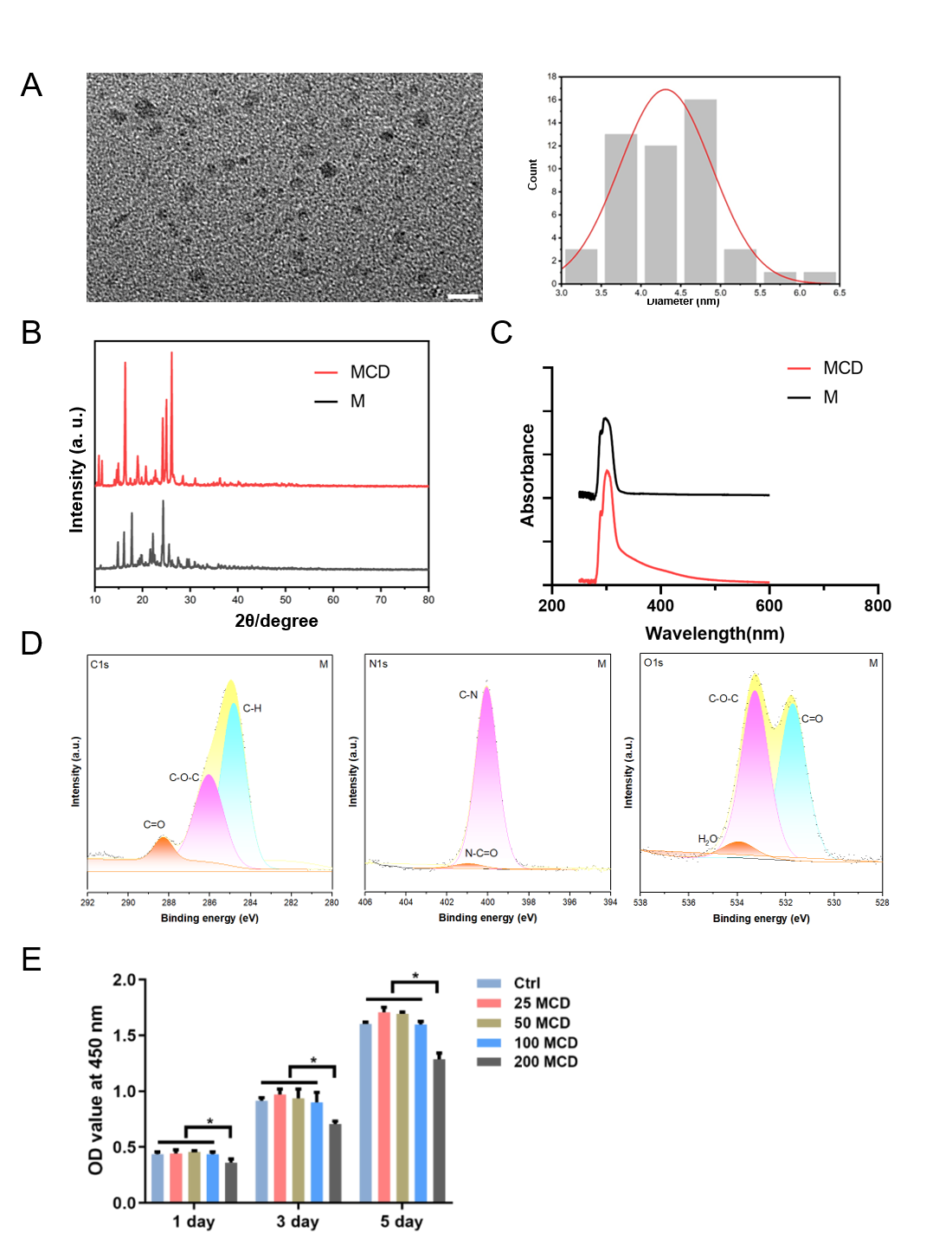
**

**Figure S1. Characterizations of the MCDs.** (**A**) Transmission electron microscopy images of MCDs and Particle size distribution map. (**B**) XRD of MCDs and melatonin. (**C**) Ultraviolet absorption spectra of MCDs and melatonin. (**D**) C1s, N1s and O1s of melatonin. (**E**) CCK-8 assay of BMSCs treated with different concentrations of MCDs. (^*^, *p* < 0.05).


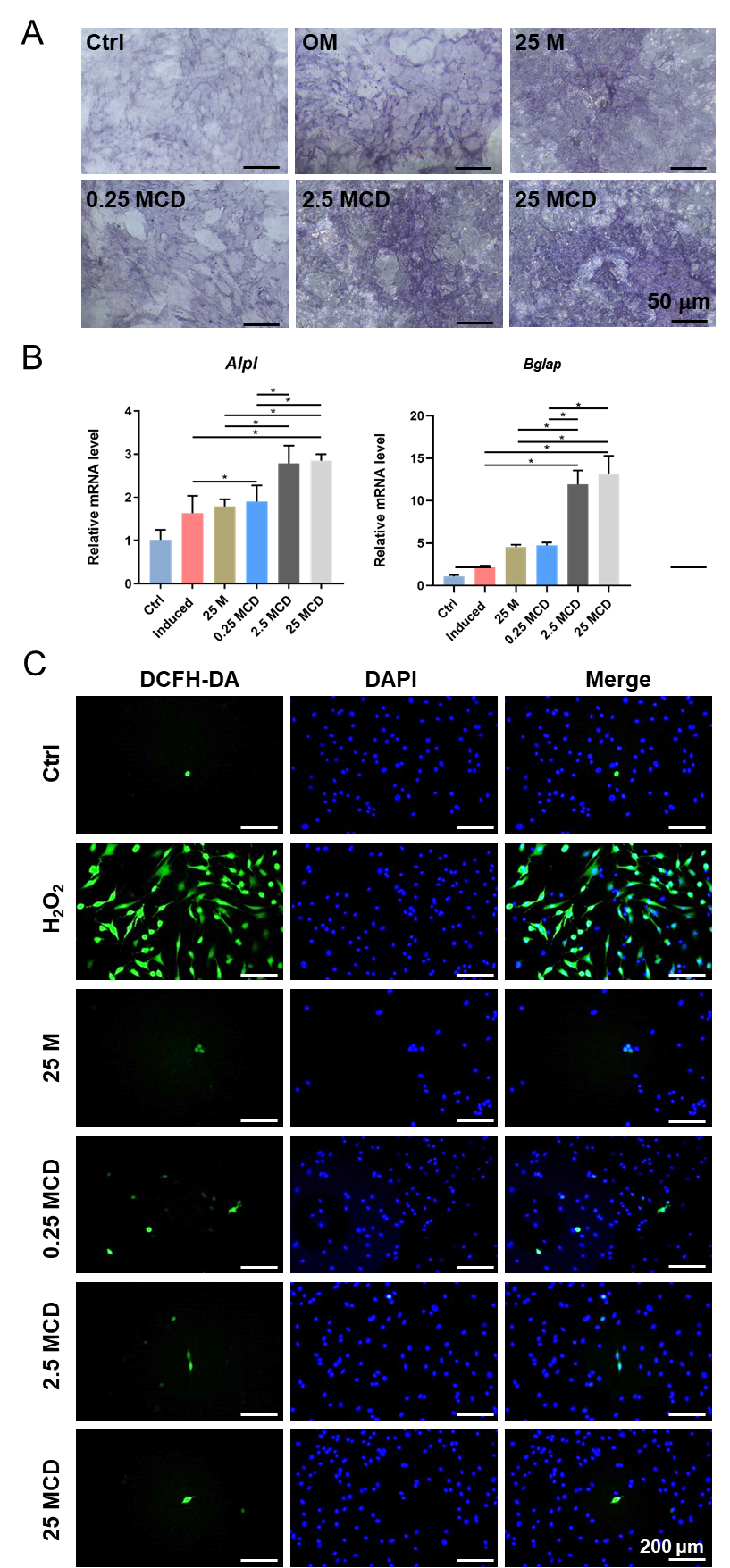


**Figure S2. *In vitro* osteogenesis and antioxidant properties of the MCDs.** (**A**) ALP staining of BMSCs treated with different concentrations of MCDs. (**B**) Expression of osteogenesis-related genes of BMSCs treated with different concentrations of MCDs. (**C**) Fluorescence images of ROS-positive BMSCs treated with H_2_O_2_. (^*^, *p* < 0.05).


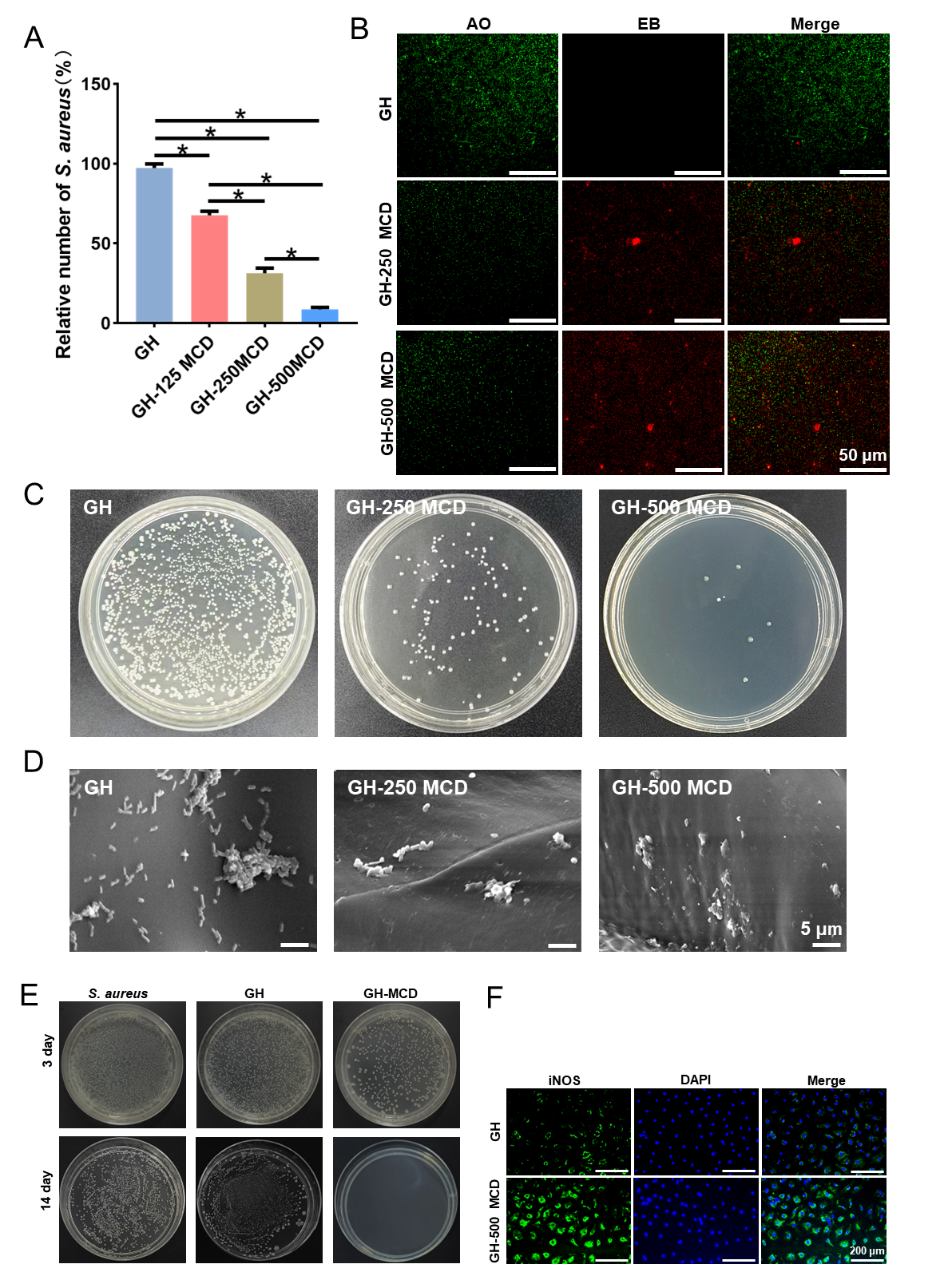


**Figure S3. Antibacterial effect of the GH-MCD composite hydrogels.** (**A**) The survival rate of *S. aureus* cultured on different hydrogels. (**B**) AO/EB staining. (**C**) Colonies of *E. coli* cultured on different hydrogels. (**D**) Morphology of *E. coli* cultured on the surface of different hydrogels. (**E**) Residual bacteria in infectious femoral condyle defects of rats. (**F**) Fluorescence images of iNOS in M1 macrophages cultured on different hydrogels. (^*^, *p* < 0.05).

**
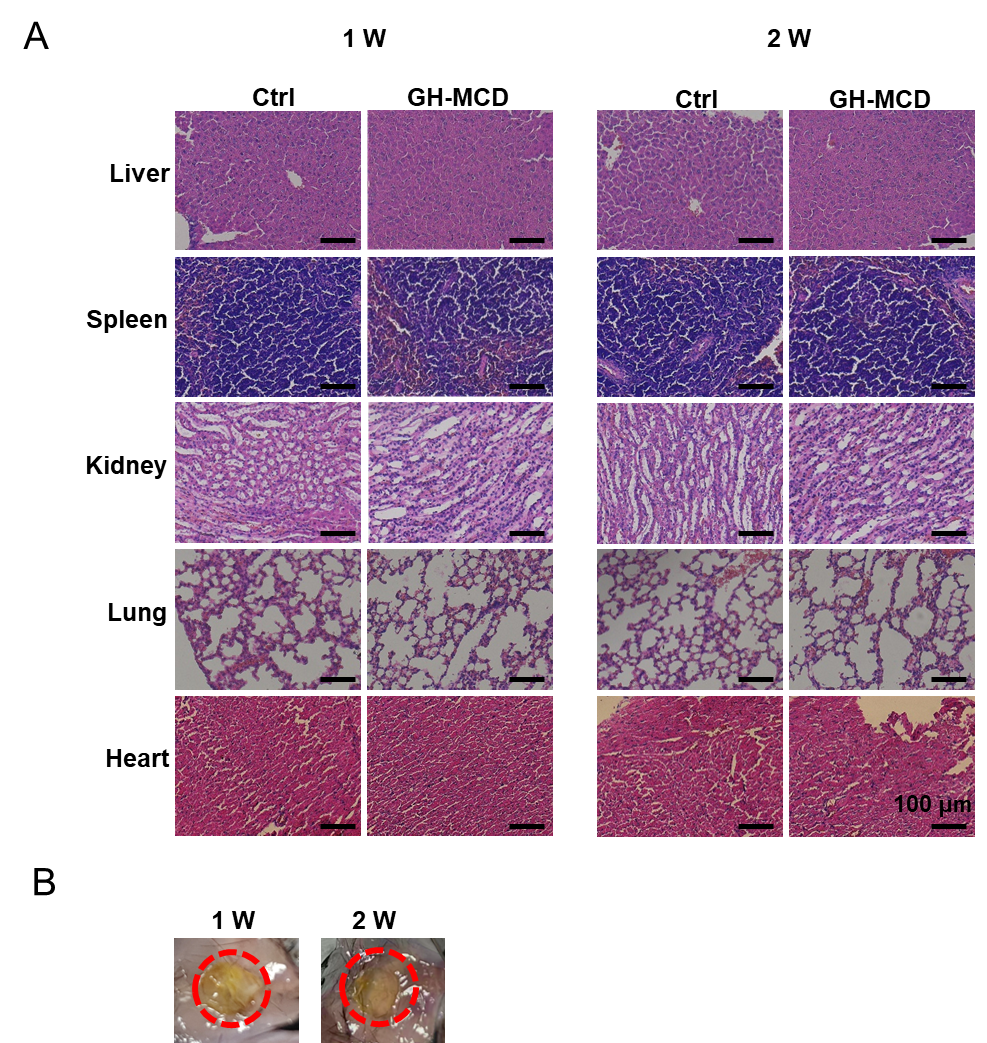
Figure S4.** ***In vivo* biosafety and stability of GH-MCD hydrogels.** (**A**) Histological evaluation of heart, liver, spleen, lung and kidney of mice at 1 and 2 weeks. (**B**) *In vivo* degradation of GH-MCD hydrogels.


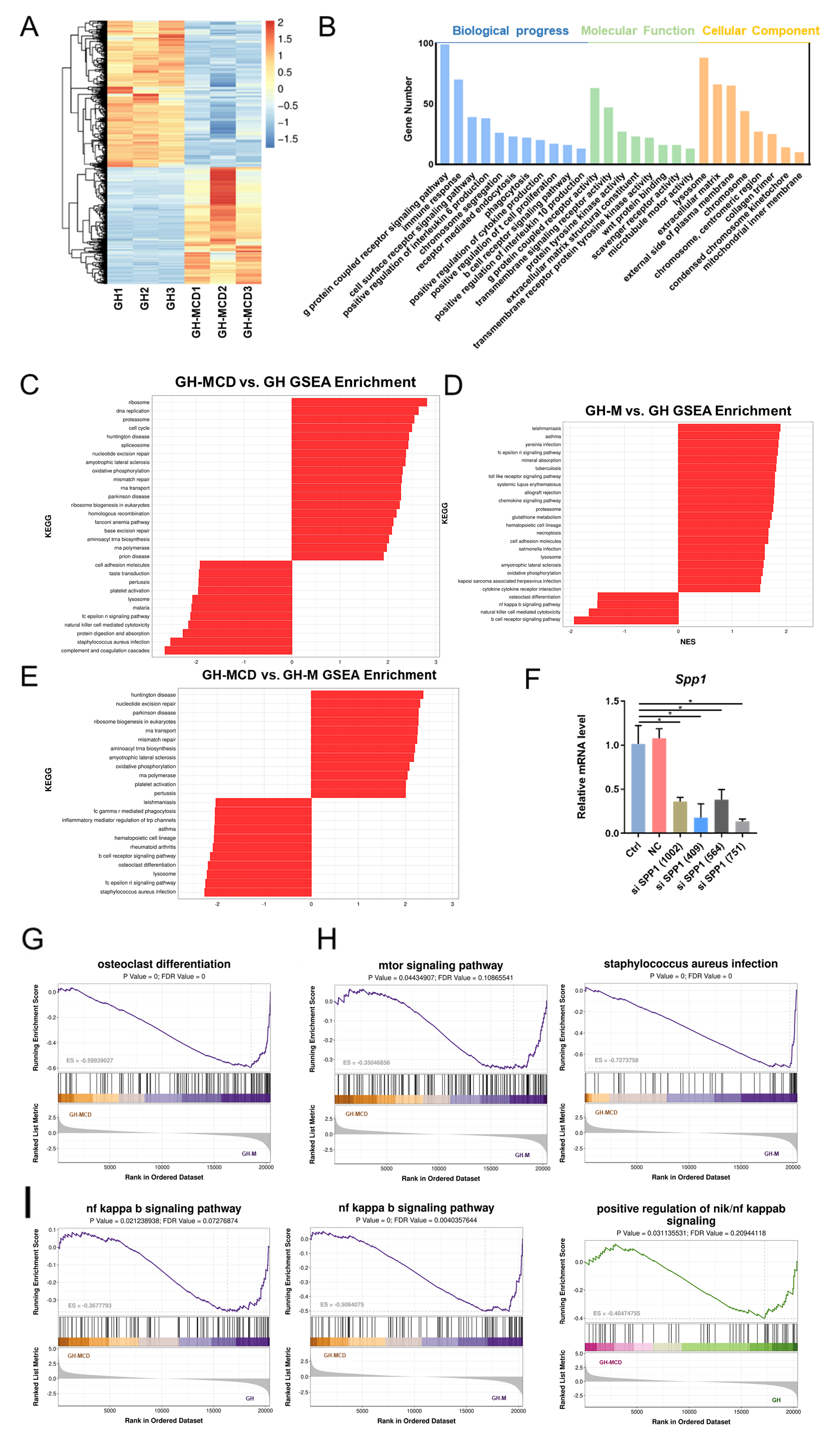


**Figure S5. Exploration of the osteogenesis mechanism of the composite hydrogels.** (**A**) Gene expression heatmap. (**B**) Gene ontology analysis. (**C**-**E**) KEGG analysis. (**F**) Relative mRNA level of *Spp1*. (**G**-**I**) Gene set enrichment analysis. (^*^, *p* < 0.05).

**Table S1. Primers and antibodies used in this study.**


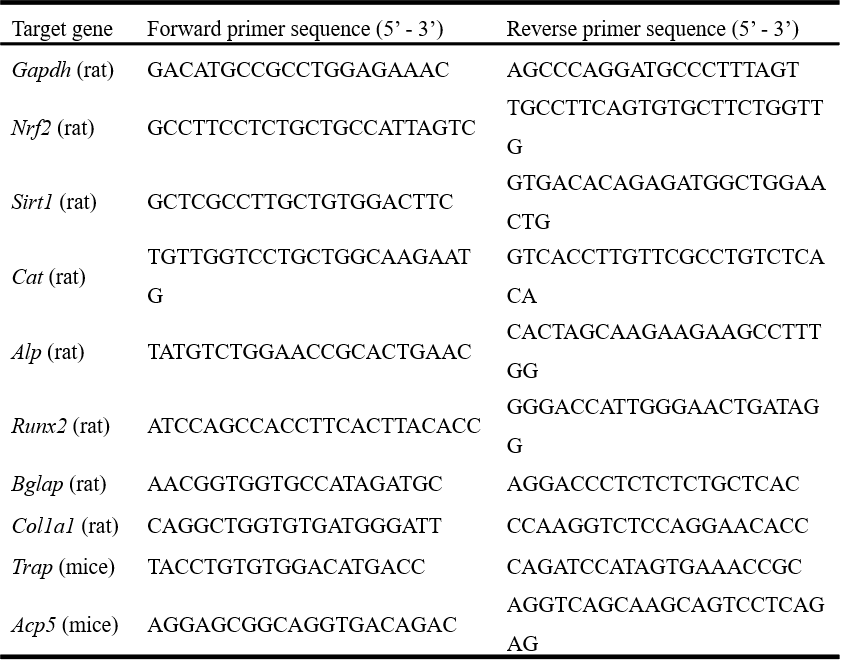


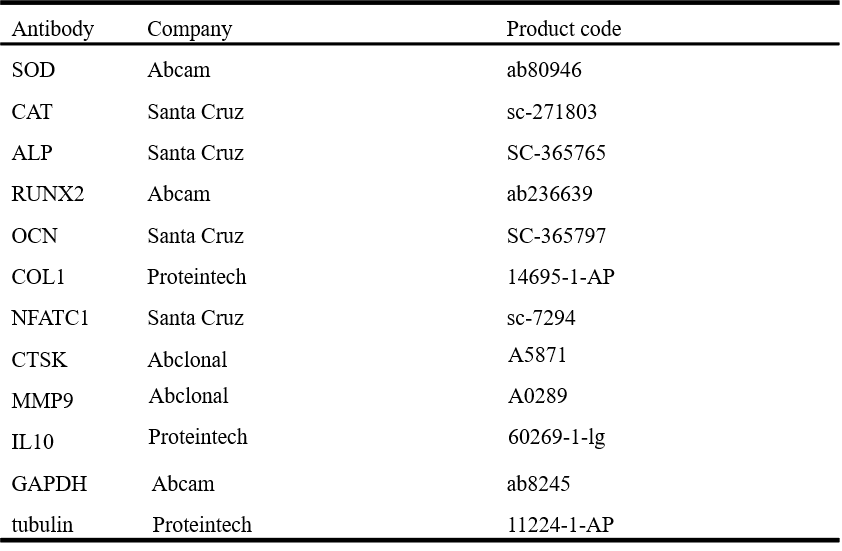

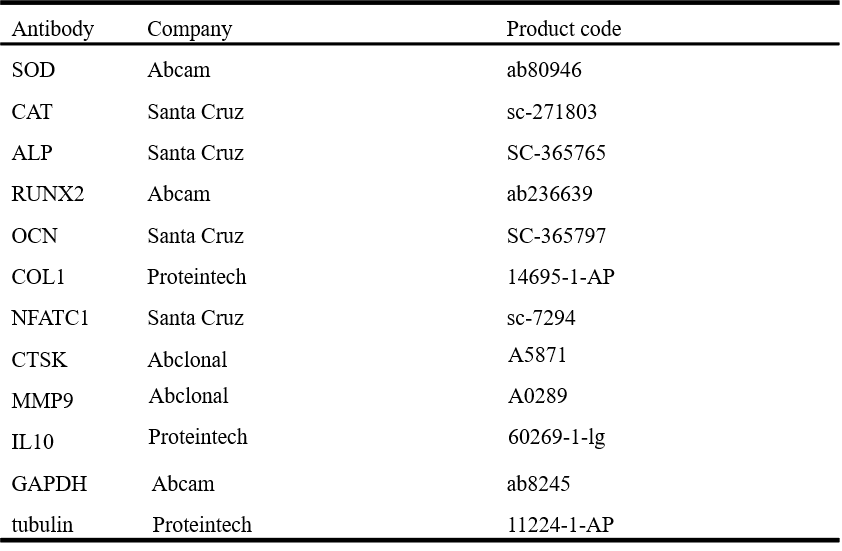


A

B
